# Architected Load Paths Enable Stiffening and Motion Programming in Soft Pneumatic Actuators

**DOI:** 10.1002/advs.77868

**Published:** 2026-09-27

**Authors:** Jie Wang, Yuwen Wu, Yuze Zhang, Ye Wei, Yuting Ma, Yingxiang Liu, Kai Guo

**Affiliations:** ^1^ School of Biomedical Engineering (Suzhou) University of Science and Technology of China Hefei People's of Republic China; ^2^ Suzhou Institute of Biomedical Engineering and Technology Chinese Academy of Sciences Suzhou People's of Republic China; ^3^ State Key Laboratory of Robotics and System Harbin Institute of Technology Harbin People's of Republic China; ^4^ Chongqing Guoke Medical Innovation Technology Development Co., Ltd Chongqing People's of Republic China

**Keywords:** architected load paths, monolithic soft actuators, motion programming, pressure‐induced stiffening, soft pneumatic actuators

## Abstract

Soft pneumatic robots exploit compliant chamber inflation to generate large and adaptive deformations, but increased pressure often produces ballooning rather than useful force or stiffness. This actuation–load‐bearing decoupling limits their use in tasks that require both controlled motion and mechanical support. Here we report MARS (Monolithic Architected Reinforcement Strategy), a single‐material soft pneumatic actuator that embeds beam–column‐inspired load paths within an unchanged external envelope. Internal lateral beams guide bending and restrain lateral expansion, whereas radial columns tether the chamber wall and suppress ballooning. Together, these load paths redirect pneumatic deformation toward ordered structural engagement during actuation. Compared with an unconstrained baseline, MARS maintained its intended morphology up to 200 kPa and showed a 5.8‐fold pressure‐induced increase in lateral stiffness. At matched bending angles, it generated 8.4‐fold higher tip force than the baseline. Selective removal of lateral beams further programmed the same monolithic template into continuous‐bending, localized hinge‐like‐bending, and torsional variants. These variants enabled crawling‐robot locomotion, load‐bearing dexterous grasping, and EEG‐guided wearable hand assistance. By coupling pneumatic actuation with internal load‐path engagement, MARS establishes an architecture‐guided strategy for mechanically supportive and motion‐programmable soft pneumatic structures.

## Graphical abstract

A single MARS template introduces an architected reinforcement strategy for programmable soft pneumatic actuators through internal structural organization and selective beam removal. This monolithic architecture enables deformation reconfiguration from continuous bending to joint‐like articulation and torsional motion, allowing one compliant platform to achieve diverse robotic functions including grasping, dexterous manipulation, and locomotion.

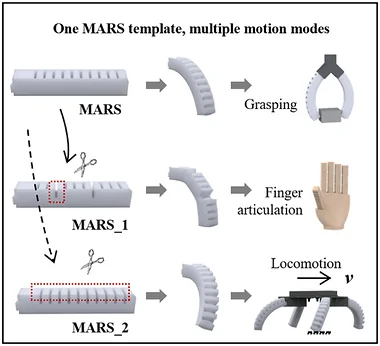

## 1 Introduction

In rigid engineering, function and mechanical performance are rarely defined by material properties alone, but are shaped by structural organization and topology [1]. Beams, columns, ribs, trusses, and their connections are organized into load‐bearing frameworks that route forces, constrain deformation, and stabilize the overall structure [2, 3]. This coordination between functional form and load‐bearing architecture has long guided the design of buildings and engineered structures [4]. Architected compliant robots and reconfigurable mechanical systems further demonstrate that relatively high‐modulus materials or assemblies of discrete rigid components can be organized to achieve shape morphing, compliant motion, tunable stiffness, and load‐bearing tasks [5, 6, 7, 8, 9].

By contrast, soft pneumatic actuators are formed from intrinsically compliant elastomers, such as silicone [10, 11, 12], and generate bending, extension, or twisting through inflation of deformable chambers while maintaining safe interaction [12, 13, 14, 15]. Yet conventional soft pneumatic actuators are often designed primarily for chamber inflation and motion generation [10, 12, 16, 17, 18], with limited internal architecture for routing loads or constraining expansion. At elevated pressures, their chambers can therefore undergo excessive ballooning or lose morphological stability [19], making additional pneumatic input difficult to convert into stable tip‐force output, shape retention, or load‐bearing stiffness development.

Existing strategies improve soft pneumatic performance through fiber reinforcement [20, 21], strain‐limiting layers [22], rigid or semi‐rigid skeletons [23, 24], external constraints, jamming structures, or variable‐stiffness modules [25, 26, 27]. These approaches have advanced soft grasping, wearable assistance, and soft locomotion, but often rely on heterogeneous reinforcement, added interfaces, or auxiliary stiffening components [23, 28, 29, 30]. Moreover, conventional strain‐limiting approaches primarily regulate strain distribution and bending direction through passive constraints, while dynamically coordinating deformation and stiffness development during actuation remains challenging. Thus, a key challenge remains: can a single‐material, intrinsically compliant pneumatic actuator synchronously generate prescribed deformation and develop load‐bearing stiffness during actuation without heterogeneous reinforcement or auxiliary stiffening components?

In this work, we present MARS, a Monolithic Architected Reinforcement Strategy for soft pneumatic actuators that embeds beam–column‐inspired load paths within a single‐material soft pneumatic body. MARS introduces lateral constraint beams and radial support columns into a baseline pneumatic actuator template while preserving the external actuator envelope. The lateral beams regulate bending evolution and restrain excessive lateral expansion, whereas the radial columns provide internal tethering. During pressurization, this architecture redirects pneumatic input from ballooning‐dominated deformation toward ordered load‐path engagement, coupling prescribed deformation with load‐bearing stiffness development and enhancing tip‐force output. This pressure‐activated interaction constitutes a deformation‐induced self‐supporting mechanism. MARS maintained its intended morphology under pressures of up to 200 kPa while exhibiting a 5.8‐fold pressure‐induced increase in stiffness relative to its initial compliant state. In matched‐angle evaluations, it further achieved up to an 8.4‐fold increase in tip‐force output relative to the unconstrained reference actuator. To realize this monolithic topology, we implemented a Sacrificial‐Material Monolithic Casting (SMMC) route that integrates the pneumatic chamber, lateral beams, and radial support columns within a single silicone body.

Beyond mechanical reinforcement, selective removal of lateral constraint beams enabled post‐defined motion programming from the same pneumatic template. Intact MARS provided continuous bending for MARS‐NeuroGlove; MARS_1 generated localized soft‐hinge‐like bending for the all‐silicone MARS‐DexHand; and MARS_2 produced spatial torsion for locomotor limbs in Tardigrade‐Bot. Together, these demonstrations show that internal load‐path reinforcement can transform a baseline pneumatic template into a mechanically supportive and motion‐programmable soft robotic building block. By integrating deformation generation, load‐bearing stiffness development, and task‐specific motion capabilities within the same compliant body, MARS provides an architecture‐guided route for reinforcing and programming soft pneumatic actuators while preserving intrinsic compliance.

## 2 Result and Discussion

### 2.1 Architected Reinforcement of Soft Pneumatic Actuators

**FIGURE 1:**
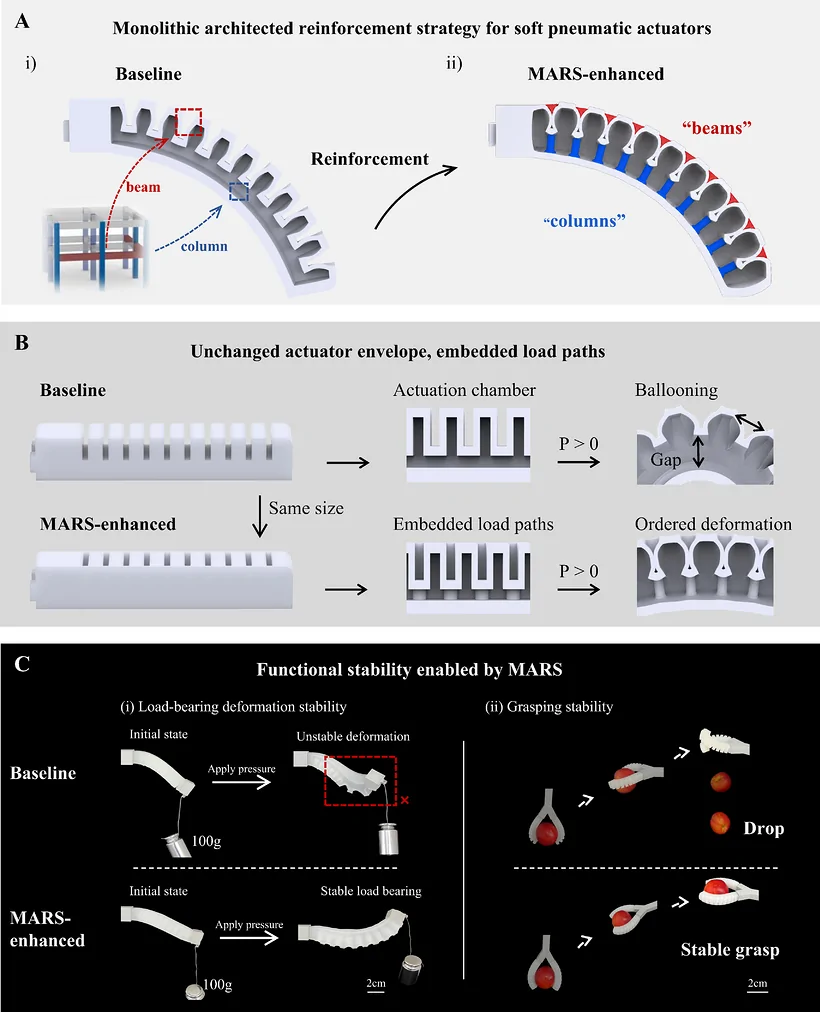
Architected reinforcement of soft pneumatic actuators. (A) Concept of MARS as an architected reinforcement strategy. A baseline pneumatic actuator is reinforced by embedding beam–column load paths, producing a MARS‐enhanced actuator with lateral beams and radial columns inside the same monolithic body. (B) Stiffening mechanism with an unchanged actuator envelope. The baseline actuator undergoes ballooning and gap formation under pressure, whereas the MARS‐enhanced actuator retains the same outer size but develops ordered deformation through embedded load paths. (C) Functional stability enabled by MARS. MARS maintains stable load‐bearing deformation and grasping, whereas the baseline actuator exhibits unstable deformation and object dropping.

MARS was developed as a monolithic architected reinforcement strategy for soft pneumatic actuators (Figure 1A). The baseline actuator serves as a pneumatic actuation template that generates bending through chamber inflation but lacks an internal load‐bearing architecture. In the MARS‐enhanced design, lateral beams and radial columns are embedded within the same silicone body, forming internal beam–column load paths without adding separate reinforcing components.

This reinforcement changes the pressure response of the actuator while preserving its external envelope (Figure 1B). In the baseline actuator, pressurization leads to chamber ballooning and gap formation, producing an unstable deformation pathway. In contrast, the MARS‐enhanced actuator retains the same outer size but contains embedded load paths. During pressurization, lateral beams restrain lateral expansion and radial columns tether the structure internally, promoting ordered deformation and load‐path engagement instead of uncontrolled ballooning.

This difference in pressure response leads to improved functional stability (Figure 1C). Under a 100 g tip payload, the baseline actuator exhibited load‐induced unstable deformation and lost its intended bending morphology, whereas the MARS‐enhanced actuator maintained a stable curved configuration and load‐bearing behavior (Figure 1C‐i). In the grasping‐stability comparison, the baseline actuator failed to retain the object, whereas the MARS‐enhanced actuator maintained a stable grasp (Figure 1C‐ii). These results show that the internal beam–column load paths improve resistance to load‐induced deformation and provide a structural basis for stable functional operation.

### 2.2 Parametric Design of the MARS Load‐Path Topology

**FIGURE 2:**
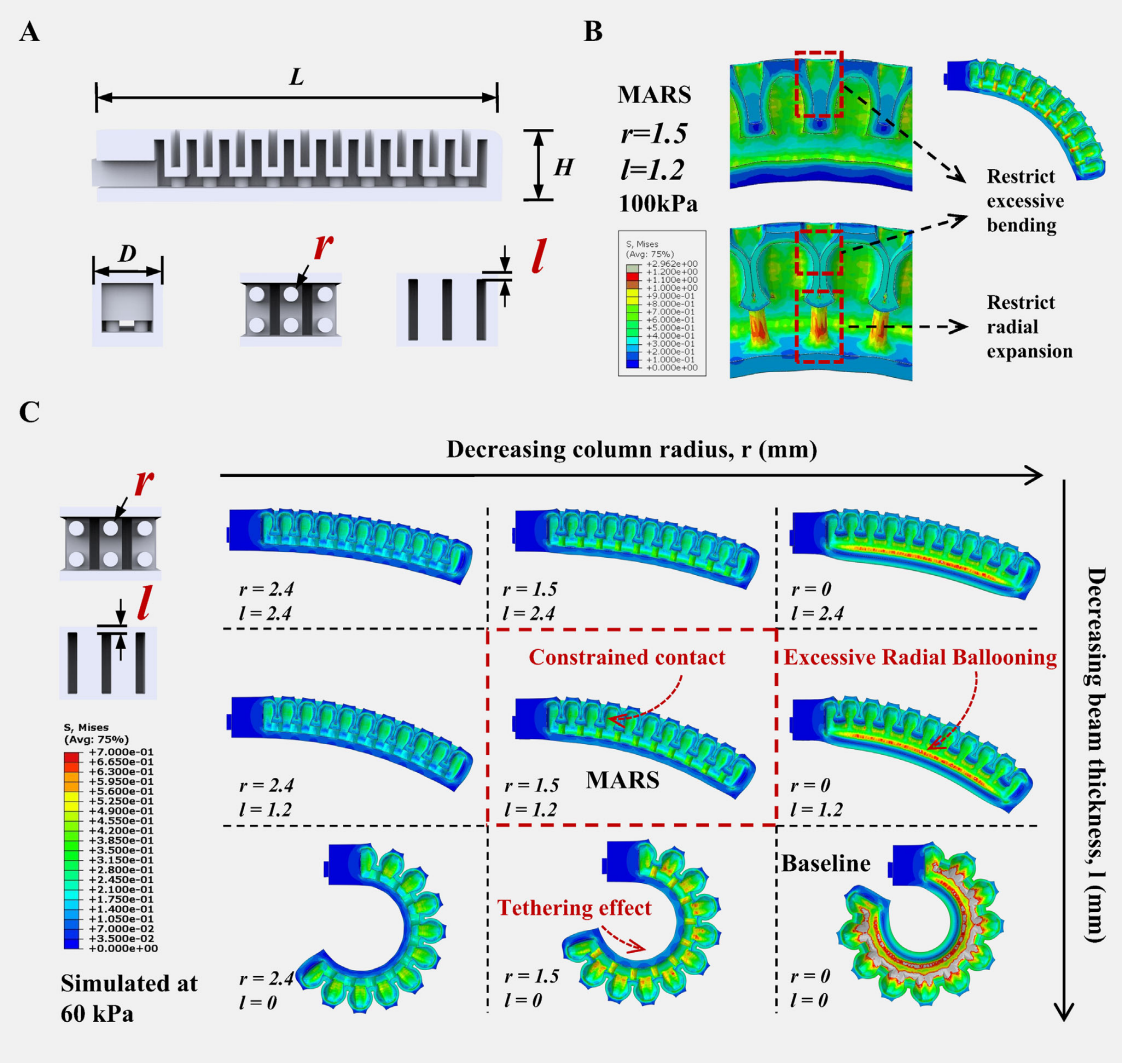
Parametric design of the MARS load‐path topology. (A) Geometric parameters of the MARS‐enhanced actuator, including the overall dimensions *L*, *H*, and *D*, and the two principal topology parameters: radial column radius *r* and lateral beam thickness *l*. (B) Representative finite‐element simulation of MARS (*r* = 1.5 mm, *l* = 1.2 mm) under 100 kPa, showing that the lateral beams restrict excessive bending and the radial columns suppress radial expansion through internal tethering. (C) Simulation matrix at 60 kPa showing how r and l regulate deformation. Reducing *r* weakens radial tethering and leads to excessive radial ballooning, whereas reducing *l* releases lateral constraint and increases bending. The selected MARS configuration balances controlled bending, radial constraint, and load‐path engagement, whereas *r* = 0 and *l* = 0 represents the unconstrained baseline actuator.

To determine how the embedded load‐path topology regulates pressure‐driven deformation, we parameterized the MARS‐enhanced actuator geometry and selected two principal topology variables: the radial column radius r and the lateral beam thickness l (Figure 2A and Figure S1). These two parameters represent the main structural functions introduced by the reinforcement strategy. The radial columns provide internal tethering that constrains radial expansion, whereas the lateral beams regulate curvature development and lateral expansion. Together, they determine how pneumatic input is redistributed within the monolithic actuator body.

Finite‐element analysis of a representative MARS‐enhanced geometry (r = 1.5 mm, l = 1.2 mm) was performed to visualize the mechanical roles of the embedded load paths (Figure 2B). At 100 kPa, the lateral beams limited excessive bending and lateral expansion, while the radial columns constrained radial ballooning through internal tethering. Their coupled action preserved a controlled bending morphology and promoted ordered load‐path engagement. This result supports the mechanism proposed in Figure 1, in which embedded load paths redirect pneumatic deformation from ballooning‐dominated expansion toward load‐bearing structural engagement.

We further varied r and l in a bivariate simulation matrix at 60 kPa to evaluate how the two topology parameters affect bending and radial expansion (Figure 2C). When both r and l were large, the actuator became over‐constrained and showed limited bending. When r was reduced to zero, radial tethering was removed, leading to excessive radial ballooning. When l was reduced to zero, lateral constraint was released, resulting in over‐bending, defined here as large bending deformation developing before sufficient load‐bearing stiffness is established (Figure S16). In contrast, the selected MARS configuration (r = 1.5 mm, l = 1.2 mm) balanced pneumatic bending, reduced radial expansion, and ordered load‐path engagement.

These simulations reveal the complementary roles of the two topology elements. Radial columns mitigate ballooning by providing internal tethering, whereas lateral beams regulate bending and lateral expansion. Their combined effect establishes a controlled deformation regime in which the actuator maintains its intended morphology and provides a mechanical basis for load‐bearing stiffness development during pressurization. Under the same pneumatic pressure of 60 kPa, FEA further showed that MARS reduced the maximum von Mises stress from 4.11 MPa in the baseline actuator to 0.49 MPa by redistributing stress through the internal load paths (Figure S16). Thus, the parameterized beam–column topology explains how the MARS reinforcement strategy converts a baseline pneumatic template into a MARS‐enhanced actuator with controlled deformation and deformation‐induced stiffening potential.

### 2.3 Fabrication and Post‐Defined Programming of the Monolithic MARS Template

**FIGURE 3:**
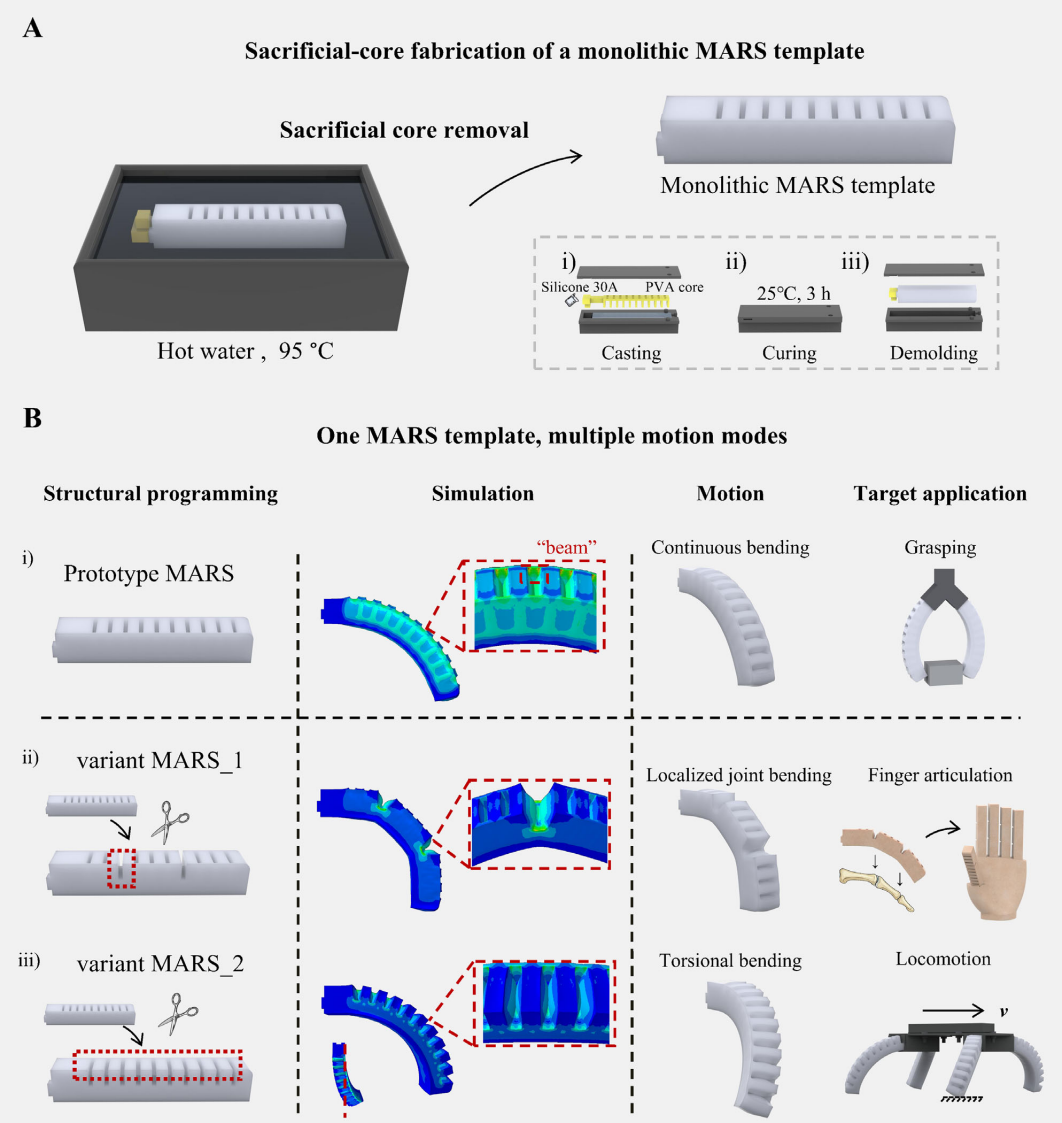
Fabrication and post‐defined programming of the monolithic MARS template. (A) Sacrificial‐core fabrication of MARS. A PVA sacrificial core was embedded during silicone casting, followed by curing, demolding, and core removal in hot water at 95°C, yielding a monolithic silicone MARS template with an integrated pneumatic chamber and internal load‐path topology. (B) One MARS template enables multiple programmed motion modes. The intact prototype MARS generated continuous bending for grasping; selective local beam removal produced MARS_1 with localized joint‐like bending for finger articulation; and asymmetric beam removal produced MARS_2 with torsional bending for locomotion.

Sacrificial‐core casting has previously been used to fabricate soft pneumatic structures with embedded internal cavities [31, 32, 33]; Here, we adapted this strategy as Sacrificial‐Material Monolithic Casting (SMMC) to fabricate a monolithic MARS template in which the pneumatic chamber and embedded beam–column load‐path architecture are continuously integrated within a single silicone body (Figure 3A). A water‐soluble PVA sacrificial core was first positioned in the mold, followed by silicone casting, curing, and demolding. During casting, the PVA core defined the interconnected pneumatic chamber, while the surrounding silicone formed the lateral beams and radial columns that remained after core dissolution. After curing, the sacrificial core was removed in hot water, leaving a monolithic silicone actuator with a continuous internal load‐path topology. This fabrication strategy provides the structural basis for implementing the MARS reinforcement topology without secondary assembly or separate reinforcing components.

The monolithic MARS template further enabled post‐defined structural programming into multiple variants and functional modes (Figure 3B). The intact template retained the original beam–column architecture and generated continuous bending, supporting grasping functions. Selective local removal of lateral beams produced MARS_1, in which deformation became concentrated in a localized hinge‐like region, enabling joint‐like bending for finger articulation. More extensive structural release produced MARS_2, which generated torsional bending and was used for locomotor functions. The simulated deformation fields were consistent with the experimentally observed motion modes (Figure S3), confirming that distinct programmed behaviors can be derived from the same monolithic template through post‐fabrication structural modification.

Together, these results show that SMMC does not merely fabricate a complex soft actuator geometry, but establishes a unified MARS template from which multiple task‐oriented variants can be derived. Thus, monolithic fabrication and post‐defined programming jointly provide the manufacturing and structural basis for translating the MARS reinforcement strategy into diversified soft robotic functions.

### 2.4 Mechanical Performance and Cyclic Stability of MARS‐Enhanced Actuators

**FIGURE 4:**
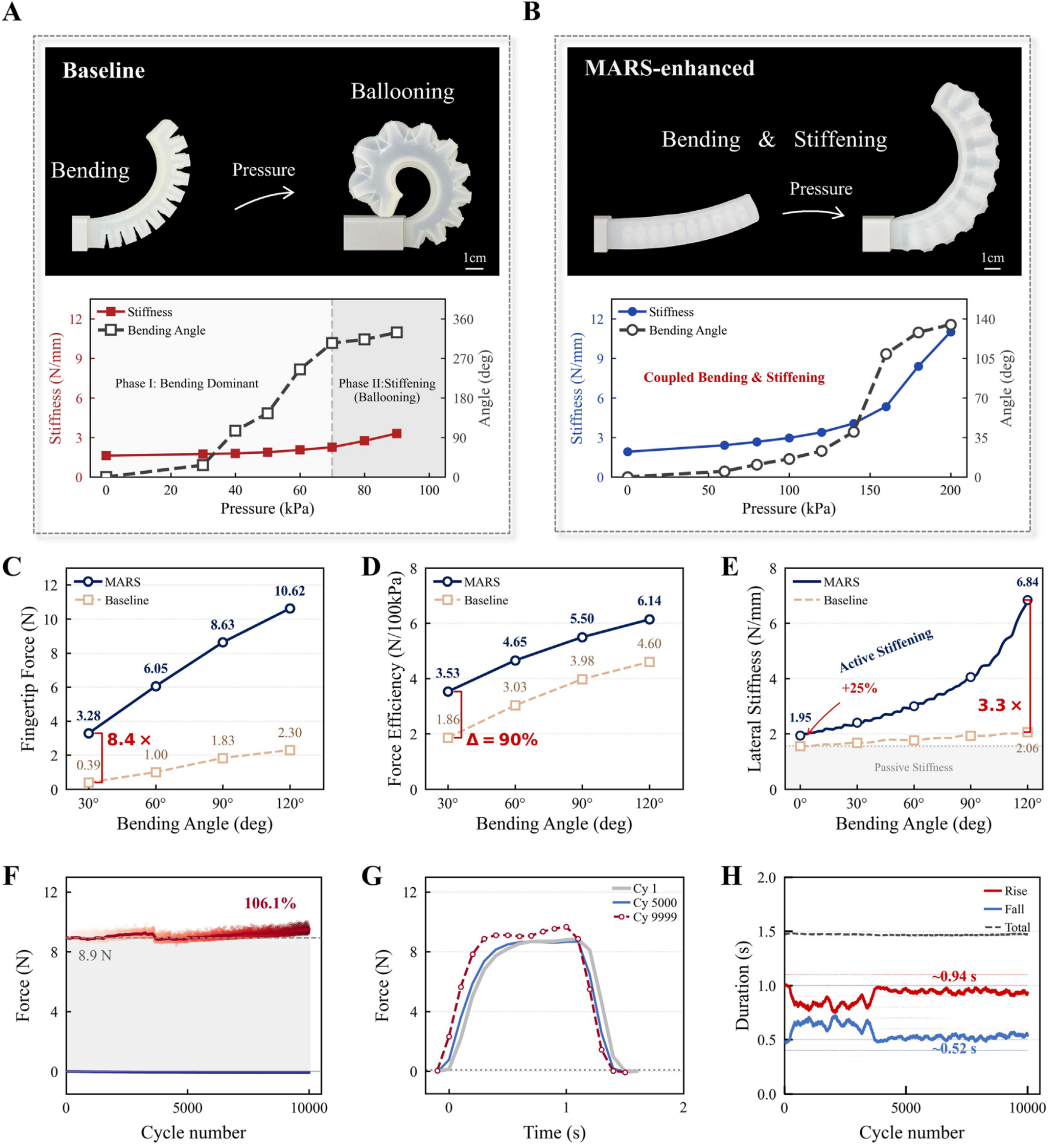
Mechanical performance and cyclic stability of MARS‐enhanced actuators. (A) Baseline actuator response under increasing pressure, showing an initial bending‐dominated phase followed by ballooning‐associated stiffening and morphological instability. (B) MARS‐enhanced actuator response under increasing pressure, showing coupled bending and stiffening through embedded load‐path reinforcement. (C) Fingertip force comparison at matched bending angles. MARS generated up to 8.4 times the fingertip force of the baseline actuator. (D) Pressure‐normalized force efficiency at matched bending angles, with MARS showing a maximum improvement of approximately 90%. (E) Lateral stiffness comparison during bending. MARS reached 6.84 N/mm at 120°, corresponding to a 3.3‐fold increase over the baseline actuator. (F to H) Cyclic stability under 160‐kPa constrained actuation for 10 000 cycles, showing stable force output, representative force–time profiles, and stable rise/fall durations.

We first examined how embedded load‐path reinforcement altered the pressure‐dependent deformation and stiffness evolution of the actuator (Figure 4A and B, Figure S9, and Movie S1). The baseline actuator exhibited a two‐stage pressure response. At low pressures, the bending angle increased rapidly, whereas lateral stiffness changed only modestly. As pressure increased beyond approximately 70 kPa, the actuator transitioned into a ballooning‐dominated regime: additional pressure mainly produced pronounced radial expansion and loss of the intended bending morphology, rather than useful load‐bearing stiffness development (Figure 4A). Thus, in the baseline actuator, pressure‐driven bending and load‐bearing stiffness development were decoupled along the loading pathway.

The MARS‐enhanced actuator followed a distinct response. At 90 kPa, it showed controlled bending without visible parasitic ballooning, and at 200 kPa it retained its intended curved morphology (Figure 4B). Across this pressure range, bending angle and lateral stiffness developed cooperatively, with lateral stiffness increasing from 1.91 N/mm in the initial compliant state to 11.02 N/mm at 200 kPa, corresponding to a 5.8‐fold pressure‐induced increase. The lateral‐stiffness measurement setup and representative probe‐contact states are shown in Figure S10. These data show that the embedded beam–column load paths redirected the pressure‐dependent deformation pathway from ballooning‐dominated instability toward coupled bending and load‐bearing stiffness development.

We next compared output performance at matched bending angles (Figure 4C). The MARS‐enhanced actuator generated higher tip‐force output than the baseline actuator throughout the tested range, reaching 3.28 N at 30° compared with 0.39 N for the baseline actuator, corresponding to an 8.4‐fold increase. Although MARS requires higher pressure to achieve the same bending angle due to the additional structural constraints, the pressure‐normalized force efficiency was further evaluated (Figure 4D). MARS exhibited an approximately 90% improvement in force efficiency at 30° bending, indicating that the enhanced force output arises from both pneumatic input and the internal load‐path architecture. Matched‐angle lateral stiffness measurements further confirmed the mechanical advantage of the embedded load‐path topology (Figure 4E). At 120° bending, the MARS‐enhanced actuator reached 6.84 N/mm, 3.3 times that of the baseline actuator (2.06 N/mm). The pressure‐dependent fingertip force of MARS was further characterized, reaching approximately 14.8 N at 200 kPa (Figure S13).

Finally, we evaluated cyclic stability using a mechanically constrained tip‐force cycling test at 160 kPa (Figure 4F to H and Figure S11). Over 10 000 cycles, the MARS‐enhanced actuator showed no measurable decline in constrained tip‐force output, with the final‐cycle force corresponding to 106.1% of the initial value. Representative force–time profiles at cycles 1, 5000, and 9999 remained comparable, without a reduction in peak output. The rise and fall durations remained stable at approximately 0.94 and 0.52 s, respectively, with a total actuation duration of approximately 1.46 s. Together, these results demonstrate that MARS reinforcement couples controlled pneumatic deformation with enhanced force output, load‐bearing stiffness development, and stable cyclic operation under mechanically constrained high‐pressure actuation.

### 2.5 MARS_2 Enables Body‐Elevated Cross‐Medium Locomotion in Tardigrade‐Bot

**FIGURE 5:**
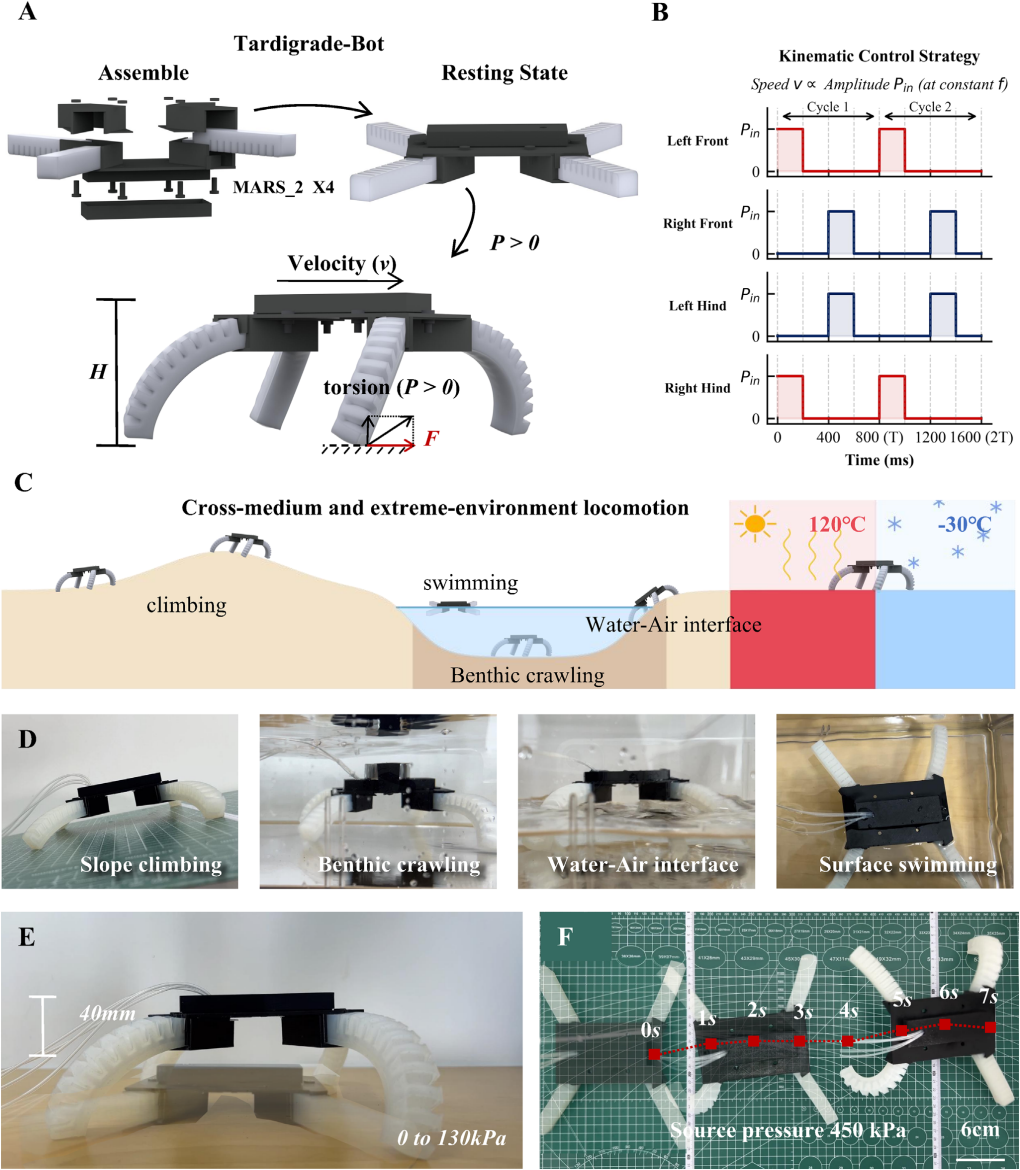
MARS_2 enables body‐elevated cross‐medium locomotion in Tardigrade‐Bot. (A) Tardigrade‐Bot was assembled using four MARS_2 actuators as compliant limbs. Upon pressurization, the limbs generated torsional deformation and ground reaction forces, lifting the body and driving forward locomotion. (B) Pulsed diagonal alternating gait. The two diagonal limb pairs were actuated alternately, and the source‐pressure setting P_in regulated limb deformation amplitude and locomotor output at a constant gait frequency. (C, D) Schematic and experimental demonstrations of cross‐medium locomotion, including slope climbing, benthic crawling, water–air interface traversal, and surface swimming, together with high‐ and low‐temperature operation. (E) Static body‐elevation test showing a 40‐mm body clearance under actuation pressures up to 130 kPa. (F) Representative terrestrial displacement sequence at P_in = 450 kPa.

To translate the torsional deformation and load‐bearing capability of MARS_2 into robot‐level locomotion, we developed a tardigrade‐inspired quadruped, Tardigrade‐Bot, using four MARS_2 actuators as compliant limbs (Figure 5A). Compared with the baseline actuator, which primarily exhibits planar bending, MARS_2 generates torsion‐enabled spatial deformation through asymmetric release of lateral beam constraints (Figure S18). This asymmetric topology introduces an out‐of‐plane motion component, enhancing 3D limb–substrate interaction and contributing to body elevation, support, and forward propulsion.

Tardigrade‐Bot was driven by a pulsed diagonal alternating gait controlled by PPC‐Bot, a Portable Pneumatic Controller configured with robot‐specific gait‐control software (Figure 5B). In each 800‐ms cycle, the left‐front/right‐hind and right‐front/left‐hind limb pairs were actuated alternately, producing a gait frequency of 1.25 Hz. At a constant gait frequency, the source‐pressure setting *P_in_* was varied to regulate limb deformation amplitude and locomotor output. Here, *P_in_* denotes the pneumatic source‐pressure setting during transient actuation rather than the equilibrated internal pressure within the MARS_2 limbs.

Using this gait, Tardigrade‐Bot demonstrated cross‐medium locomotion, including slope climbing, benthic crawling, traversal of the water–air interface, and surface swimming (Figure 5C and D and Movie S2). The robot also retained locomotor function under high‐ and low‐temperature conditions at 120°C and −30°C (Figure 5C and Movie S2). In a static body‐elevation test, the MARS_2 limbs maintained a lifted posture with a body clearance of approximately 40 mm under actuation pressures up to 130 kPa (Figure 5E). A representative locomotion sequence further showed terrestrial displacement under pulsed actuation at P_in = 450 kPa (Figure 5F). Together, these results show that MARS_2‐derived limbs convert topology‐defined torsion and load‐bearing support into body‐elevated cross‐medium locomotion.

**FIGURE 6:**
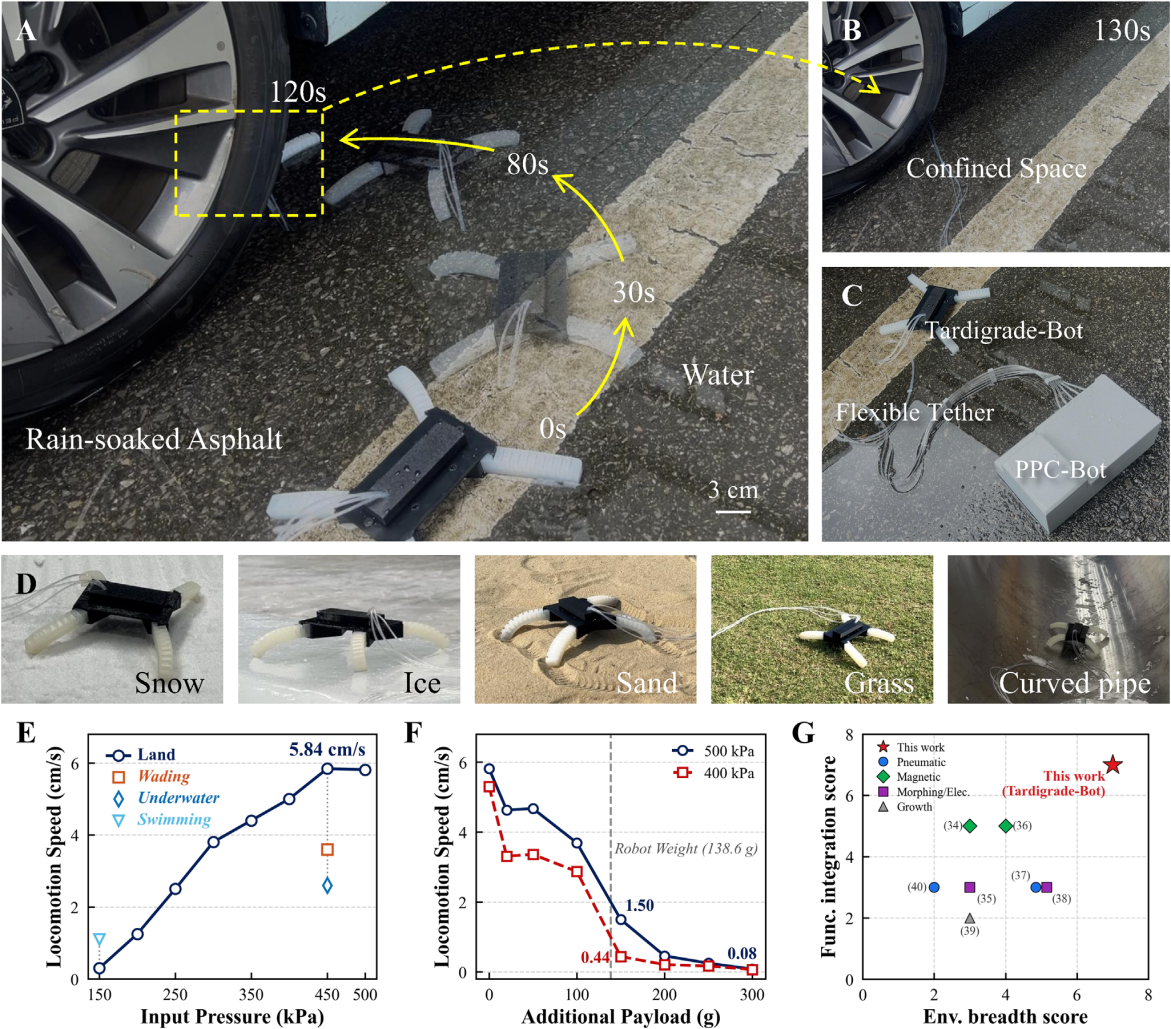
Portable deployment, multi‐terrain locomotion, and functional comparison of Tardigrade‐Bot. (A, B) Outdoor tethered locomotion on rain‐soaked asphalt and entry into a confined region adjacent to a vehicle wheel. (C) Portable pneumatic operation using PPC‐Bot, a Portable Pneumatic Controller configured with robot‐specific gait‐control software, connected through a flexible pneumatic tether. (D) Representative locomotion tests on snow, ice, sand, grass, and curved pipe‐like surfaces. (E) Locomotion speed as a function of source‐pressure setting on land, together with speeds measured during wading, underwater locomotion, and surface swimming. (F) Payload‐dependent locomotion speed at source‐pressure settings of 400 and 500 kPa; the dashed line indicates the robot mass of 138.6 g. (G) Deployment–function comparison with representative soft locomotor robots based on experimentally demonstrated environmental breadth and functional integration. Scoring criteria and literature sources are provided in Table S2.

We next evaluated portable tethered deployment outside controlled laboratory settings (Figure 6A–D and Movies S3–S6). Connected to PPC‐Bot through a flexible pneumatic tether, Tardigrade‐Bot moved on rain‐soaked asphalt with surface water and entered a confined region adjacent to a vehicle wheel. Additional continuous‐video experiments demonstrated locomotion on snow, ice, sand, grass, and curved pipe‐like substrates, as well as recovery from limb obstruction in grass. These tests establish portable tethered operation across wet, slippery, granular, vegetated, curved, and confined environments, rather than fully untethered autonomy.

Quantitative measurements characterized speed modulation and payload‐bearing locomotion (Figures 6E and F and Movie S7). On land, locomotion speed increased from 0.30 cm/s at P_in_ = 150kPa to 5.84 cm/s at P_in_ = 450kPa and remained similar atP_in_ = 500kPa, indicating a plateau at high source‐pressure settings. The robot also achieved speeds of 3.60 cm/s during wading and 2.60 cm/s during underwater locomotion at P_in_ = 450kPa, and 1.10 cm/s during surface swimming at P_in_ = 150kPa. At P_in_ = 500kPa, Tardigrade‐Bot moved at 1.50 cm/s while carrying an additional payload of 150 g, exceeding its own mass of 138.6 g. It remained measurably mobile under a maximum additional payload of 300 g, equivalent to 2.16 times its body mass, although speed decreased to 0.08 cm/s.

Finally, we compared Tardigrade‐Bot with representative soft locomotor robots [34, 35, 36, 37, 38, 39, 40] using an evidence‐based deployment–function map rather than a speed ranking, because locomotion speed depends strongly on robot scale, actuation mechanism, medium, substrate, and tethering condition (Figure 6G and Table S2). Within the selected comparison set, Tardigrade‐Bot integrated body‐elevated movement, cross‐medium locomotion, multi‐terrain operation, confined‐space traversal, recovery‐oriented locomotion, thermal‐condition testing, payload‐bearing movement, and portable tethered control within one platform. Together, these results show that MARS_2‐derived limbs translate topology‐defined spatial torsion and load‐bearing capability into integrated locomotor functions across diverse media, terrains, and deployment conditions.

### 2.6 MARS_1 Enables High‐Load Compliant Manipulation in MARS‐DexHand

**FIGURE 7:**
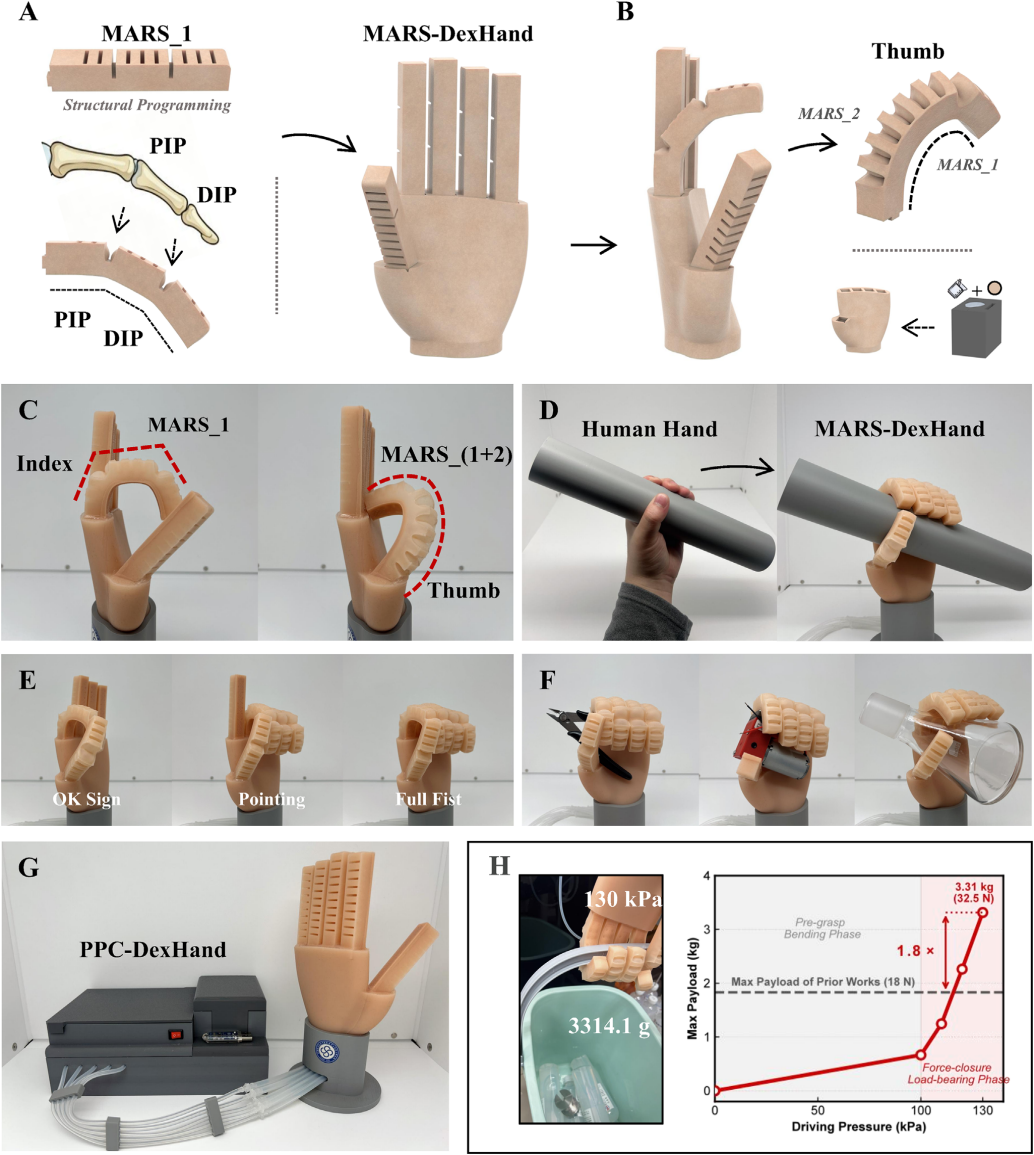
MARS_1 enables high‐load compliant manipulation in MARS‐DexHand. (A) MARS‐DexHand was assembled from MARS_1‐programmed digits, in which selective local beam removal produced PIP‐ and DIP‐like hinge‐like flexion. (B) The thumb combined MARS_1‐derived flexion and MARS_2‐derived out‐of‐plane deformation to enable opposition. (C) Experimental demonstrations of MARS_1 index‐finger articulation and hybrid MARS_1–MARS_2 thumb motion. (D) Oppositional grasping of a cylindrical object by a human hand and MARS‐DexHand. (E and F) Representative hand postures and object‐grasping demonstrations. (G) Portable pneumatic operation using PPC‐DexHand. (H) Suspended‐payload test showing that MARS‐DexHand supported 3314.1 g, corresponding to approximately 32.5 N, at 130 kPa.

Soft robotic hands enable compliant grasping, but high‐load manipulation often depends on rigid skeletons, hybrid structures, or jamming‐based reinforcement strategies [23, 41, 42]. To examine whether post‐defined MARS variants can be assembled into a compliant manipulator with both dexterous motion and load‐bearing capability, we developed MARS‐DexHand, an anthropomorphic soft hand with an all‐silicone hand body (Figure 7A and Figure S12). Four fingers were constructed from MARS_1 variants, in which local removal of lateral beams produced localized joint‐like deformation corresponding to PIP‐ and DIP‐like flexion (Figure 7A). This intentional constraint release sacrifices part of the mechanical support of intact MARS (Figure S19) to achieve joint‐like articulation compatible with human finger kinematics. The thumb was designed by combining MARS_1 and MARS_2‐derived functions, enabling both bending and out‐of‐plane opposition (Figure 7B). In this way, finger articulation and thumb opposition were achieved through post‐defined structural programming, without rigid finger skeletons, tendon transmissions, or heterogeneous reinforcing components.

We next characterized the resulting motion behaviors at both the digit and hand levels. The MARS_1 index finger showed localized joint‐like bending, while the hybrid MARS_(1+2) thumb generated oppositional motion suitable for grasp formation (Figure 7C). At the hand scale, MARS‐DexHand conformed around a cylindrical object and formed an oppositional grasp configuration comparable to that of a human hand (Figure 7D and Movie S8). Under multichannel pneumatic actuation, the hand further generated representative anthropomorphic postures, including an OK sign, pointing, and a full fist (Figure 7E). It also grasped objects with different geometries and functional requirements, including pliers, an air pump, and a transparent cup (Figure 7F). Integration with PPC‐DexHand further demonstrated compatibility with a portable pneumatic control platform (Figure 7G).

Finally, we evaluated load‐bearing grasping using a suspended‐payload test (Figure 7H). At a driving pressure of 130 kPa, MARS‐DexHand supported a payload of 3314.1 g, corresponding to approximately 32.5 N, while maintaining a fully soft, all‐silicone architecture. The pressure–payload curve revealed two sequential regimes: an initial pre‐grasp bending phase, in which increasing pressure mainly drove finger closure, followed by a force‐closure load‐bearing phase after stable object engagement. Together, these results show that post‐defined MARS variants can be integrated into an anthropomorphic all‐silicone soft hand that combines joint‐like articulation, thumb opposition, object‐adaptive grasping, portable pneumatic actuation, and substantial load‐bearing capability.

### 2.7 Intact MARS Enables SSVEP‐Guided Wearable Hand Assistance in MARS‐NeuroGlove

Soft robotic gloves have been explored for wearable hand assistance and post‐stroke rehabilitation, and SSVEP‐based brain–computer interfaces have provided command pathways for soft robotic glove training [28, 43, 44, 45]. Beyond gripper‐type manipulation and anthropomorphic hand demonstrations, we integrated five intact MARS modules into MARS‐NeuroGlove to explore the rehabilitation‐oriented application of MARS‐based soft actuation.

**FIGURE 8:**
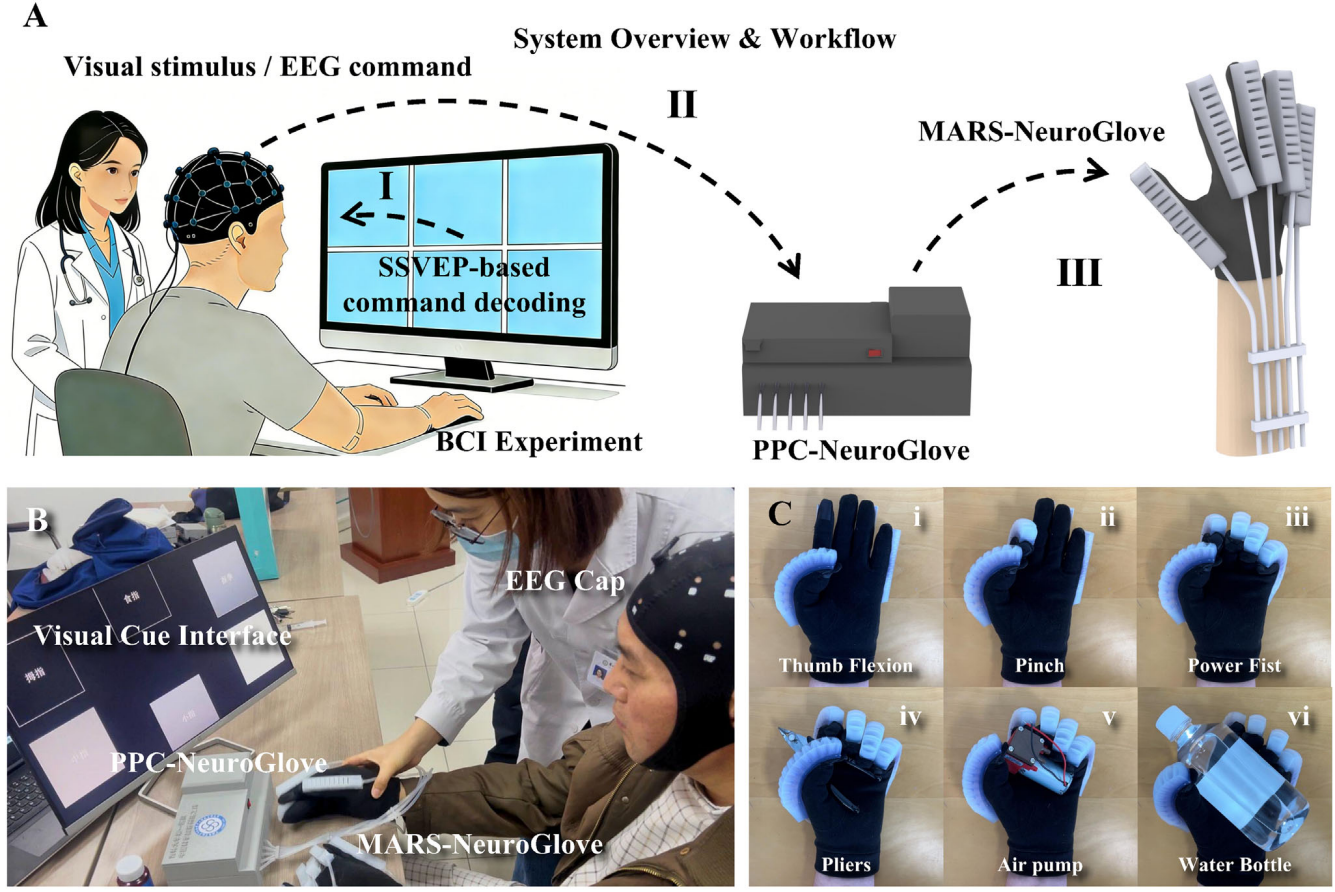
Intact MARS enables SSVEP‐guided wearable hand assistance in MARS‐NeuroGlove. (A) Workflow of MARS‐NeuroGlove, integrating a visual‐cue/EEG command interface, SSVEP‐based command decoding, PPC‐NeuroGlove, and a wearable glove driven by five intact MARS modules. (B) Clinical‐site feasibility demonstration, showing the participant wearing an EEG cap and MARS‐NeuroGlove while interacting with the visual cue interface. (C) Assisted hand motions and object interactions, including thumb flexion, pinching, power‐fist closure, and grasping pliers, an air pump, and a water bottle.

MARS‐NeuroGlove connects a visual‐cue/EEG command interface with PPC‐NeuroGlove and a wearable glove containing five intact MARS modules (Figure 8A). Intact MARS was selected because it preserves the complete beam–column load‐path topology, enabling continuous bending with mechanically supportive actuation for assisted finger flexion. In a clinical‐site demonstration, the participant wore an EEG cap and MARS‐NeuroGlove while interacting with the visual cue interface, and decoded SSVEP commands triggered pneumatic assistance through PPC‐NeuroGlove (Figure 8B and Movie S10).

The glove further performed representative assisted motions and object interactions, including thumb flexion, pinching, power‐fist closure, and grasping a pair of pliers, an air pump, and a water bottle (Figure 8C and Movie S9). These demonstrations show that intact MARS modules can support externally driven continuous hand flexion and functional multi‐finger assistance in a wearable format. This clinical‐site feasibility demonstration suggests that MARS provides a mechanically capable soft actuation platform for future rehabilitation‐oriented evaluation, without establishing therapeutic efficacy in the present study.

## 3 Conclusion

MARS introduces a monolithic architected reinforcement strategy for soft pneumatic actuators by placing load‐bearing topology inside a single compliant body. In contrast to conventional chamber‐dominated designs, MARS uses lateral beams and radial columns to route pneumatic deformation through internal load paths. This architecture redirects inflation from parasitic ballooning toward coupled bending, shape retention, and stiffness development. At the actuator level, MARS retained its intended morphology at 200 kPa and showed a 5.8‐fold pressure‐induced increase in lateral stiffness. It also generated up to 8.4‐fold higher tip force than the baseline actuator at matched bending angles. These results suggest that internal load‐path engagement can convert pneumatic input into useful mechanical support without changing the external actuator envelope.

This strategy differs from existing stiffness‐enhancement or variable‐stiffness mechanisms [8, 46, 47, 48, 49, 50] in three main aspects. First, reinforcement is encoded within a single silicone body rather than introduced through heterogeneous materials, external skeletons, or auxiliary stiffening modules. Second, stiffening develops during pneumatic actuation through pressure‐activated load‐path engagement. Third, the same reinforced template can be post‐programmed by selective constraint release. Although some reported systems achieve larger stiffness‐change ratios, they often require additional material phases, interfaces, or system‐level components. MARS instead emphasizes material homogeneity, internal structural organization, and actuation‐coupled stiffness development (Table S1).

The transferability of this design principle was illustrated using three MARS‐derived robotic platforms. Intact MARS supported continuous bending for wearable hand assistance, MARS_1 produced localized joint‐like bending for load‐bearing grasping, and MARS_2 generated torsional deformation for body‐elevated locomotion. These demonstrations show that functional diversity can be obtained by reconfiguring internal constraint pathways within the same pneumatic template. Thus, MARS does not rely on stacking independent modules to create new functions. Instead, it translates actuator‐level topology design into task‐oriented robotic motion and support.

The current strategy also has several limitations. First, the embedded beam–column topology increases the pressure required to reach a target deformation, which places higher demands on pneumatic supply, response speed, and portable control. Second, the present post‐defined programming method relies on selective beam removal and is therefore irreversible. Third, the complex internal channels make complete dissolution of the PVA sacrificial core slow, typically requiring 1 to 2 days. Future work should also quantify sample‐to‐sample variability, fabrication success rate, leakage resistance, and long‐term durability across independently fabricated actuators. Addressing these issues will be important for translating MARS from proof‐of‐concept prototypes to robust soft robotic systems.

Overall, MARS highlights a route for designing soft pneumatic systems through internal topology rather than material hybridization alone. By treating load paths, constraint distributions, and local release patterns as design variables, compliant pneumatic bodies can be engineered to coordinate deformation, support, and motion programming. This approach may extend to bending, extension, twisting, continuum, and multichamber pneumatic structures. Recent advances in architected metamaterials, constraint‐programmed pneumatic actuators, multimodal soft robotic systems, and rehabilitation‐oriented soft robotics further highlight the broad potential of structural and functional programming for tailoring mechanical properties, deformation modes, and application‐specific robotic functions [51, 52, 53, 54]. More broadly, it suggests that architected soft materials can serve not only as deformable bodies, but also as mechanically organized platforms for programmable soft machines.

## 4 Experimental Section

### 4.1 Fabrication of MARS

MARS actuators were fabricated using a sacrificial‐material‐based monolithic casting process (SMMC). Silicone Rubber Skin 30 elastomer (Shenzhen Guoyuan Technology Co., Ltd., China) was used as the actuator material. Water‐soluble PVA filament and PLA filament (Bambu, Shenzhen Tuozhu Technology Co., Ltd., China) were used to print the sacrificial cores and molds, respectively, using a commercial FDM 3D printer (Bambu H2D, Shenzhen Tuozhu Technology Co., Ltd., China). Before casting, the PVA sacrificial core was fixed in the mold. The two components of Silicone Rubber Skin 30 were mixed at a 1:1 ratio, vacuum‐degassed for 40 s, poured into the mold, and cured at 25°C for 2 h. After demolding, the actuator was immersed in 95°C water for 1 to 2 days to dissolve the PVA sacrificial core, leaving a monolithic silicone actuator with an integrated pneumatic cavity, lateral constraint beams, and internal support columns.

Baseline control actuators were fabricated using the same process as MARS, except that the PVA sacrificial core lacked the column‐forming features. After molding, all lateral constraint beams were manually removed to obtain the topology‐free Baseline actuator.

Additional fabrication states, sacrificial‐core configurations, and prototype comparisons are shown in Figures S4–S6.

### 4.2 Fingertip‐Force Test of MARS

Fingertip‐force measurements were performed using an HZC‐T force sensor with a 0–30 N range (Bengbu Chengying Sensor Co., Ltd., China). Before each test, the force sensor was zero‐calibrated, and force data were recorded at 10 Hz. The actuator was fixed in a custom fixture, with the actuator tip positioned above the force sensor. Pneumatic pressure was applied stepwise, and the reported fingertip forces were calculated as the average values over the steady‐state force plateau after each pressure input.

### 4.3 Lateral‐Stiffness Test of MARS

For lateral‐stiffness testing, a thin rod was laterally pressed into the side wall of the actuator by a fixed displacement of 2 mm, and the opposite end of the rod was connected to an HZC‐T force sensor. After the 2‐mm indentation was applied, pneumatic pressure was increased stepwise, and the lateral reaction force was recorded at 10 Hz after zero calibration. The lateral stiffness was calculated as the measured lateral reaction force divided by the indentation depth.

### 4.4 Cyclic Durability Test of MARS

Cyclic durability was evaluated by repeating constrained fingertip‐force tests for 10 000 cycles at 160 kPa. Each cycle consisted of 1200 ms inflation and 800 ms deflation.

### 4.5 FE Simulations

Finite‐element simulations were performed in Abaqus using a second‐order Yeoh hyperelastic model fitted from uniaxial tensile‐test data of Silicone Rubber Skin 30. The fitted material parameters were C_10_ = 0.1224 and C_20_ = 0.0016 (Figure S2). The actuator body was discretized using free tetrahedral meshing with tetrahedral solid elements. One end of the actuator was fully constrained to reproduce the experimental clamping condition, and pneumatic pressure was applied to the internal chamber surfaces. Frictionless surface‐to‐surface self‐contact was assigned to the external surfaces of adjacent actuation units that came into contact during pressurization. The representative MARS model used an internal support‐column radius of r = 1.5mm and a lateral constraint‐beam thickness of l = 1.2mm. The Baseline model retained the same overall pneumatic actuator geometry but removed the internal support columns and lateral constraint beams, corresponding tor = 0 and l = 0.

The finite‐element simulations were used to analyze the deformation mechanism and structural effects of the embedded beam–column architecture. Direct comparisons between experimental and simulated pressure‐dependent deformation are provided in Figure S20.

### 4.6 Application‐Specific Integration of MARS‐Based Robotic Systems

All MARS‐based robotic systems were driven by a portable pneumatic controller (PPC) platform with a shared hardware architecture, including a microcontroller, relay modules, solenoid valves, pneumatic tubing, and an air‐supply unit. Application‐specific control programs were configured as PPC‐Bot, PPC‐DexHand, and PPC‐NeuroGlove for locomotion, grasping, and wearable assistance, respectively.

Tardigrade‐Bot was assembled from four MARS_2 actuators mounted on a high‐temperature‐resistant polycarbonate (PC) body frame. Opposite limb pairs were prepared with left‐ or right‐side lateral‐beam removal to generate mirrored torsion‐driven trajectories and were connected to PPC‐Bot for programmed diagonal‐gait locomotion.

MARS‐DexHand consisted of four MARS_1 actuators, one hybrid MARS_1–MARS_2 actuator, and an all‐silicone palm with embedded pneumatic tubes. The actuators were bonded to the palm using silicone adhesive, and the palm casting process is shown in Figure S12.

MARS‐NeuroGlove was assembled by bonding five intact MARS actuators onto a wearable glove using silicone adhesive. The actuators were connected to PPC‐NeuroGlove for steady‐state visual evoked potential (SSVEP)–guided hand assistance.

### 4.7 Human Participant and Ethical Approval

The MARS‐NeuroGlove feasibility demonstration involving a participant with hemiparesis was approved by the Medical Ethics Committee of Suzhou New District People's Hospital (approval no. 2024–076).

### 4.8 Experimental Design and Statistical Analysis

Unless otherwise stated, experiments were conducted once using a single actuator or prototype (n = 1). Reported curves represent individual measurements, and no inferential statistical tests or error bars were applied. Steady‐state force values were averaged over the corresponding recording interval. The durability test comprised 10 000 consecutive cycles on the same actuator, rather than independent replicates. No data were excluded.

## Author Contributions

Conceptualization: **Jie Wang**, **Kai Guo**. Methodology: Jie Wang, Kai Guo, **Ye Wei**. Investigation: Jie Wang, **Yuze Zhang**. Visualization: Jie Wang, **Yuwen Wu**. Funding acquisition: Kai Guo. Project administration: Kai Guo, **Yingxiang Liu**. Supervision: **Yuting Ma**, Yingxiang Liu, Kai Guo. Writing – original draft: Jie Wang, Kai Guo, Yuting Ma. Writing – review & editing: Jie Wang, Kai Guo, Yuwen Wu, Yuting Ma, Yuze Zhang, Yingxiang Liu, Ye Wei.

## Funding

National Key R&D Program of China (2023YFB4706200). The Innovative Key Project of Suzhou Institute of Biomedical Engineering and Technology, the Chinese Academy of Sciences (Grant No. CX20260103). State Administration of Foreign Experts Affairs of China (Grant No. H20240225). The pilot projects for fundamental research in Suzhou(Grant No. SSD2023014), Natural Science Foundation Project of Chongqing (Grant No. 2024NSCQ‐MSX0007). Science and Technology Development Plan Project of Jilin Province (Grant No. 20240305049YY, No. 20260301020YY).

## Conflicts of Interest

The authors declare that they have no competing interests.

## Supporting information




**Supporting File 1**: advs77868‐sup‐0001‐SuppMat.pdf.

**Supporting File 2**: advs77868‐sup‐0002‐SuppMat.docx.

**Supporting File 3**: advs77868‐sup‐0003‐MovieS1.mp4.

**Supporting File 4**: advs77868‐sup‐0004‐MovieS2.mp4.

**Supporting File 5**: advs77868‐sup‐0005‐MovieS3.mp4.

**Supporting File 6**: advs77868‐sup‐0006‐MovieS4.mp4.

**Supporting File 7**: advs77868‐sup‐0007‐MovieS5.mp4.

**Supporting File 8**: advs77868‐sup‐0008‐MovieS6.mp4.

**Supporting File 9**: advs77868‐sup‐0009‐MovieS7.mp4.

**Supporting File 10**: advs77868‐sup‐0010‐MovieS8.mp4.

**Supporting File 11**: advs77868‐sup‐00011‐MovieS9.mp4.

**Supporting File 12**: advs77868‐sup‐00012‐MovieS10.mp4.

**Supporting File 13**: advs77868‐sup‐00013‐MovieS11.mp4.

## Data Availability

The data that support the findings of this study are available in the supplementary material of this article.
