# Supplementary material for: Architected Load Paths Enable Stiffening and Motion Programming in Soft Pneumatic Actuators
Source: Adv Sci (Weinh). 2026 Sep 27:e77868. Online ahead of print. doi: 10.1002/advs.77868 (PMC13616343; doi:10.1002/advs.77868)
Supplement: Supplementary file 1 [file ADVS-9999-e77868-s002.pdf]

Supplementary Materials

The PDF file includes:

Figs. S1 to S20
Tables S1 and S2

Other Supplementary Material for this manuscript includes the following:

Movies S1 to S11

Theoretical model of pressure-induced bending and force output in MARS actuators

The MARS actuator schematically shown in Supplementary Fig. S1 is modeled as a soft continuum beam with periodically distributed pneumatic chambers, lateral constraint beams, internal support columns, and an asymmetric bottom constraint. Upon pressurization, the chamber roof and sidewalls sustain distributed pressure loads, while the internal load-bearing topology constrains radial expansion and modifies the effective bending stiffness. Because the upper chambered region and the lower substrate are structurally asymmetric, the resultant pressure force is offset from the neutral axis, generating an effective bending moment and producing one-way bending.

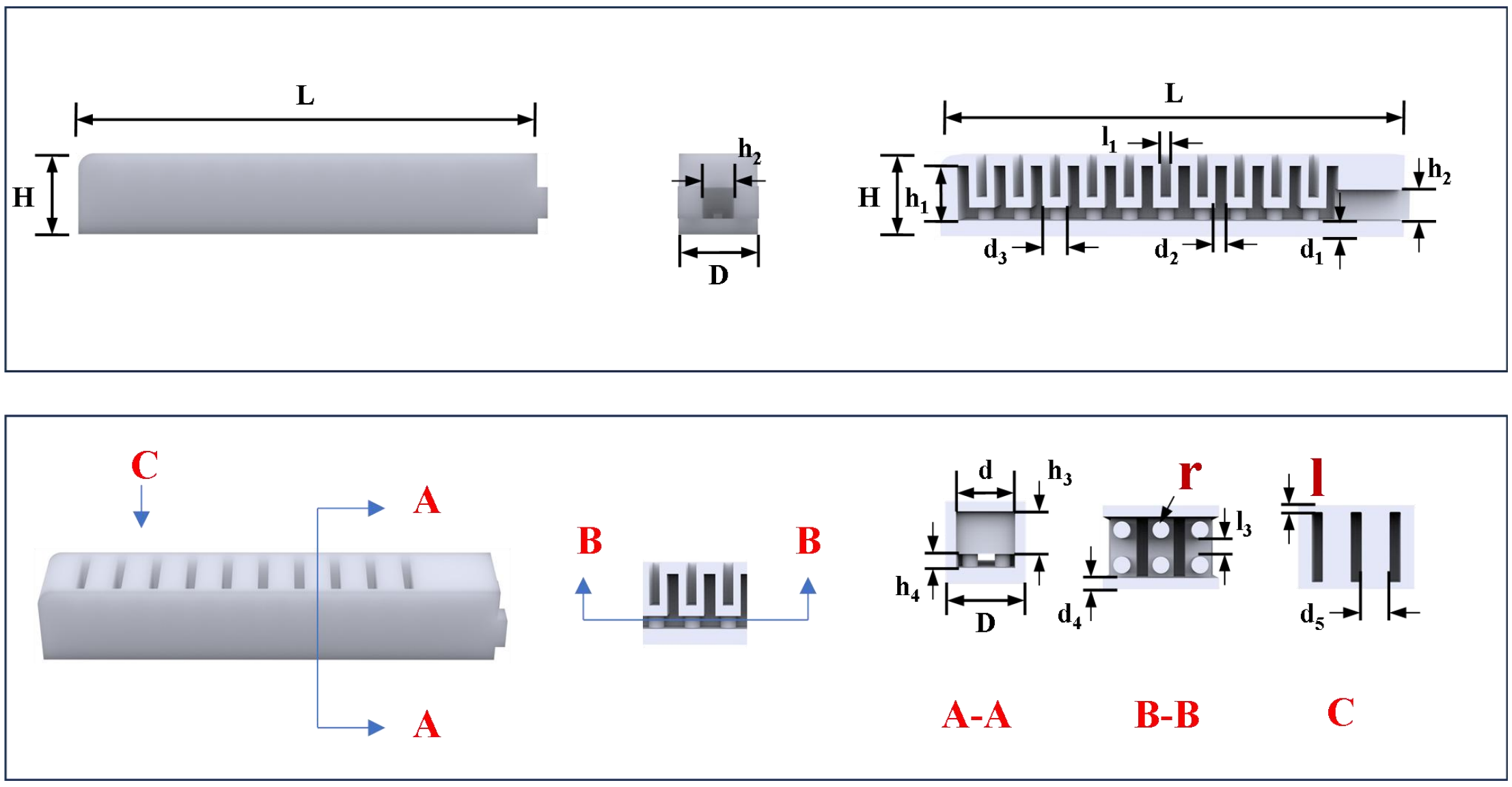

Fig. S1. Geometric parameters and cross-sectional definitions of the MARS actuator used in the theoretical model.

(1) Geometric Parameters

L	Total length of the MARS actuator
H	Total height of the MARS actuator
D	Total width of the MARS actuator
d	Effective chamber length in the longitudinal direction
d_1	bottom thickness
d_2	Effective active length
d_3	Distal passive length
d_4	Thickness of side groove
d_5	Spacing between local basal grooves
h_1	Main chamber height
h_2	Top constraint layer thickness
h_3	Height of Upper cavity
h_4	Height of Lower cavity and internal support columns

l	Sidewall thickness
l_1	Rib thickness between neighboring chambers internal support columns
l_3	Spacing between the internal support columns
r	Radius of internal support columns

(2) Mechanical and Actuation Parameters

P	Input positive pressure
E_t	Equivalent tangent modulus of the material
ν	Poisson's ratio
$\bar{y}$	Equivalent neutral-axis position
e	Eccentric distance between pressure resultant and neutral axis
I_{eff}	Effective second moment of area
κ	Curvature
θ	Total bending angle
F_{out}	Output force
F_{tip}	Tip force / blocked force
x_c	Distance from the contact point to the fixed end
η_p	Pressure transmission efficiency factor
η_I	Local geometric correction factor

(3) Pressure-Induced Driving Moment

For a single chamber, the positive pressure P mainly acts on the effective roof area. Because the chamber extends across the width direction, the effective pressure area can be approximated as

$$A_{\mathrm{eff}}=\eta_p dD$$

where η_p is a pressure-transmission efficiency factor accounting for rounded corners, transition regions, local bulging, and three-dimensional deformation nonuniformity. The equivalent pressure force of one chamber is then

$$F_p=PA_{\mathrm{eff}}=\eta_p PdD$$

Since the line of action of the pressure resultant is offset from the equivalent neutral axis by an eccentric distance e, the effective driving moment of one chamber is

$$M_c=F_p e=\eta_p PdDe$$

For the present structure, the eccentric distance can be approximated as

$$e=\mathrm{h}_4+\frac{\mathrm{h}_1}{2}-\bar{y}$$

Each chamber has an effective longitudinal dimension d, and the rib thickness between neighboring chambers is l_1. Therefore, the effective number of chambers in the active segment can be approximated by $n\approx\frac{d_2}{d+l_1}$.

Where $\mathrm{h}_4+\frac{\mathrm{h}_1}{2}$ is the centroid position of the main chamber measured from the bottom, and $\bar{y}$ is the equivalent neutral-axis position. Accordingly, the total driving moment over the active segment is

$$M_p=nM_c=\frac{d_2}{d+l_1}\eta_p PdDe$$

which can be rearranged as

$$M_p=\eta_p PDe\frac{dd_2}{d+l_1}$$

This expression shows that the driving moment increases with P, D, e, d, and d_2, while it decreases with the chamber pitch $d+l_1$.

(4) Equivalent Bending Stiffness

The bending response is jointly determined by the pressure-induced driving moment and the structural bending stiffness. The actuator is approximated as an outer rectangular beam of width D and height H, with the main chamber treated as an equivalent void. The influence of local grooves and connecting ribs is incorporated through correction terms.

The chamber occupancy ratio along the longitudinal direction is defined as

$$\phi=\frac{d}{d+l_1}$$

Thus, the equivalent void area of the main chamber in the homogenized cross-section is

$$A_v = \phi D h_1$$

The area of the outer envelope is

$$A_0 = DH$$

With the bottom as the reference, the centroid of the outer envelope is located at $H/2$, whereas the centroid of the main chamber is located at

$$y_v = h_4 + \frac{h_1}{2}$$

Hence, the equivalent neutral-axis position is

$$\bar{y} = \frac{A_0 \frac{H}{2} - A_v y_v}{A_0 - A_v}$$

The second moment of area of the outer envelope about its own centroid is

$$I_0 = \frac{DH^3}{12}$$

and that of the main chamber is

$$I_v = \frac{\phi D h_1^3}{12}$$

Accordingly, the baseline equivalent second moment of area is

$$I_b = \left(I_0 + A_0 \left(\frac{H}{2} - \bar{y} \right)^2 \right) - (I_v + A_v (y_v - \bar{y})^2)$$

Because the present structure also contains local features represented by h_3, h_4, d_4, d_5, l, l_3, r, which further perturb the actual stiffness, a local geometric correction factor η_I is introduced, yielding

$$I_{\mathrm{eff}} = \eta_I I_b$$

The effective bending stiffness is therefore

$$(EI)_{eff} = E_t I_{eff}$$

where E_t is the equivalent tangent modulus of Shore 30A silicone in the working strain range.

(5) Pressure-Bending Relationship

Under quasi-static conditions, the pressure-induced driving moment is balanced by the elastic restoring moment:

$$M_p = (EI)_{\mathrm{eff}} \kappa$$

Substituting the above expressions gives

$$\kappa = \frac{\eta_p P D e}{E_t I_{\mathrm{eff}}} \cdot \frac{d d_2}{d + l_1} \cdot \frac{1}{d_2} = \frac{\eta_p P d D e}{(d + l_1) E_t I_{\mathrm{eff}}}$$

Accordingly, the total bending angle over the active segment is

$$\theta=\kappa d_2=\frac{\eta_p PdDed_2}{(d+l_1)E_t I_{\text{eff}}}$$

Thus, in the moderate-pressure regime, the bending angle varies approximately linearly with pressure. Under the constant-curvature assumption, the tip coordinates are

$$x_{\text{tip}}=\frac{\sin\theta}{\kappa}\ ,\ y_{\text{tip}}=\frac{1-\cos\theta}{\kappa}$$

For small bending angles, the transverse tip displacement can be approximated as

$$y_{\text{tip}}\approx\frac{\kappa d_2^2}{2}$$

(6) Output Force and Blocked-Force

When the finger contacts an external object, part of the driving moment maintains the deformed posture, and the remaining part is converted into output force. Let x_c be the distance from the contact point to the fixed end. Static moment balance gives

$$M_p=E_t I_{\text{eff}}\frac{\theta}{d_2}+F_{\text{out}}x_c$$

Hence, the general output force is

$$F_{\text{out}}=\frac{\eta_p PDe\frac{dd_2}{d+l_1}-E_t I_{\text{eff}}\frac{\theta}{d_2}}{x_c}$$

For a fully blocked tip, one may take $\theta\approx0$ and $x_c=d_2$, leading to the tip/blocked force

$$F_{\text{tip}}=\frac{\eta_p PdDe}{d+l_1}$$

Therefore, under the blocked condition, the tip force is mainly governed by the pressure P, chamber size d, actuator width D, eccentricity e, and the chamber pitch $d+l_1$.

(7) Effects of Structural Parameters

- **Pressure *P***

$$\theta\propto P,\ \kappa\propto P,\quad F_{\text{tip}}\propto P$$

Increasing P will increases both bending curvature and force.

- **Chamber size *d***

Increasing d enlarges the pressure area and usually increases both bending and force, although excessive d may reduce η_p because of severe bulging.

- **Actuator width *D***

A larger D directly increases the pressure resultant and is therefore more beneficial for force generation than for bending amplification.

- **l_1**

$$\theta,\ \kappa,\ F_{\text{tip}}\propto\frac{1}{d+l_1}$$

An increase in l_1 will decrease the density of the air cavity per unit length,

thereby reducing the driving capability, but it will contribute to enhancing structural stability.

- h_1

Increasing h_1 usually enlarges the eccentricity e and reduces stiffness, thereby enhancing bending, although excessive depth may deteriorate structural stability.

- h_2

A thicker top layer suppresses chamber expansion and increases stiffness, thus reducing bending sensitivity.

- h_4

A thicker bottom layer increases the constraint effect and stiffness, suppressing free bending while improving force-transmission stability.

- h_3, d_4, d_5, l, l_3, r

These parameters primarily affect performance through η_I and η_p: wider local grooves d_4, d_5 usually reduce stiffness and increase bending, whereas larger wall thicknesses l, l_3 increase stiffness and reduce bending. A larger fillet radius r is generally beneficial for reducing stress concentration and improving durability.

(8) Model Summary

The MARS actuator can be modeled as a pressure-induced bending moment acting on an asymmetric soft beam. The governing relationships are

$$\theta=\frac{\eta_p PdDed_2}{(d+l_1)E_t I_{\text{eff}}}$$

$$F_{\text{tip}}=\frac{\eta_p PdDe}{d+l_1}$$

Different structural dimensions affect the actuator through two coupled pathways: one by changing the driving moment, and the other by modifying the effective bending stiffness

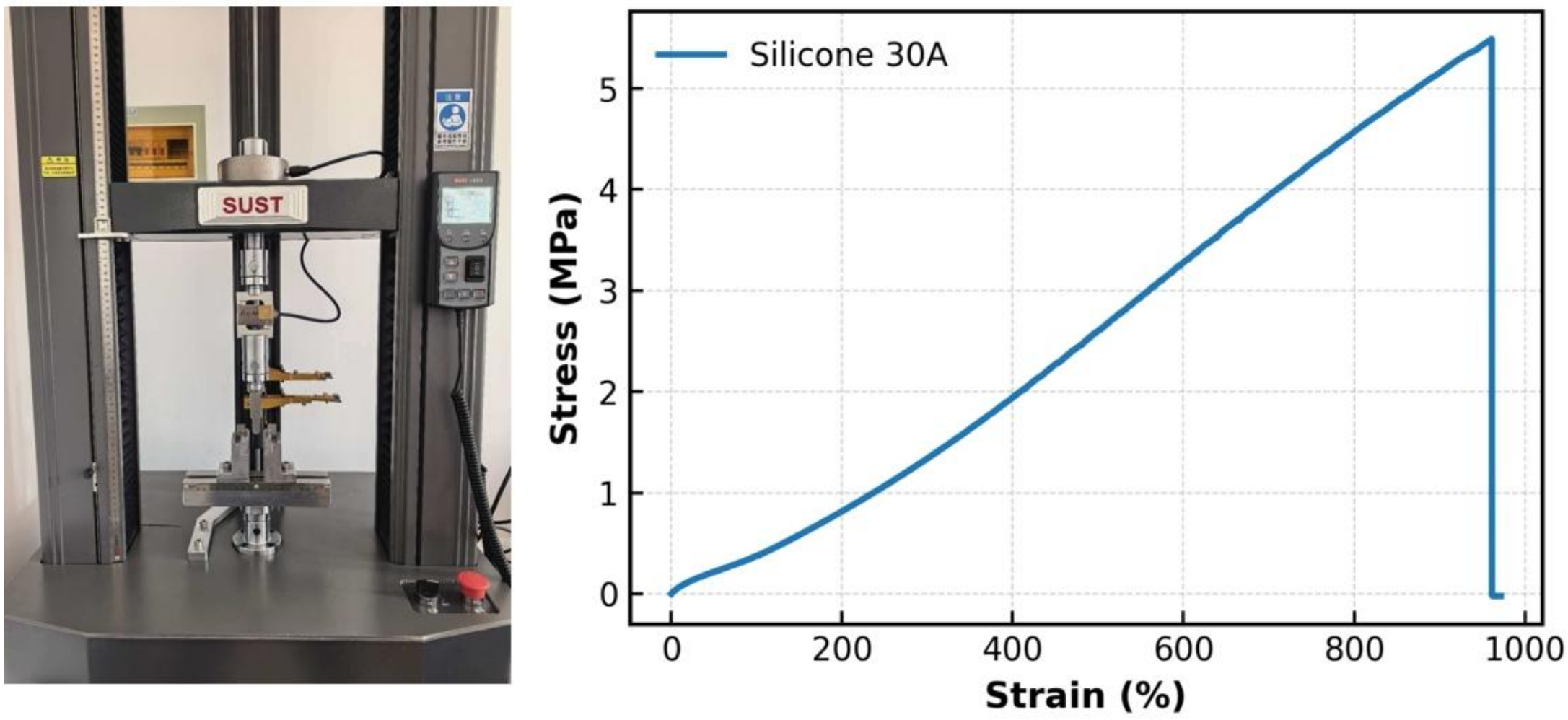

Fig. S2. Material characterization of Silicone Rubber Skin 30. A standard silicone strip with a thickness of 2 mm and a width of 8 mm was tested under uniaxial tension. The resulting stress-strain curve was used to determine the hyperelastic material parameters for finite-element simulations.

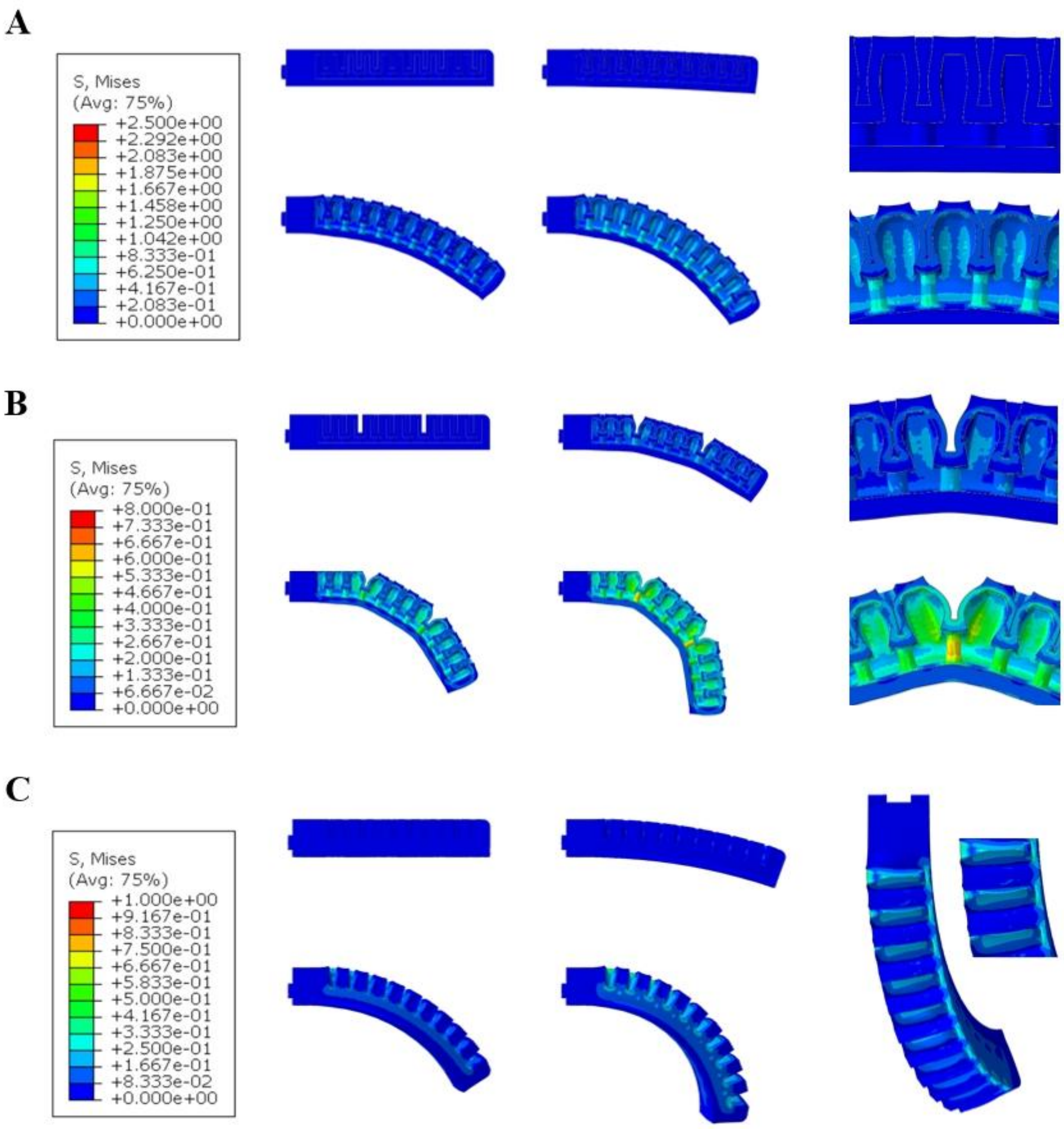

Fig. S3. Simulated deformation modes and stress distributions of MARS variants. (A) Intact MARS shows continuous bending under pneumatic pressurization. (B) MARS_1, generated by local removal of lateral constraint beams, shows localized joint-like bending. (C) MARS_2, generated by asymmetric removal of lateral constraint beams, shows torsion-dominant spatial deformation. The enlarged panels show local structural deformation and von Mises stress distributions in the corresponding regions. These simulations support the post-fabrication structural programming strategy used to generate task-specific MARS motion modes.

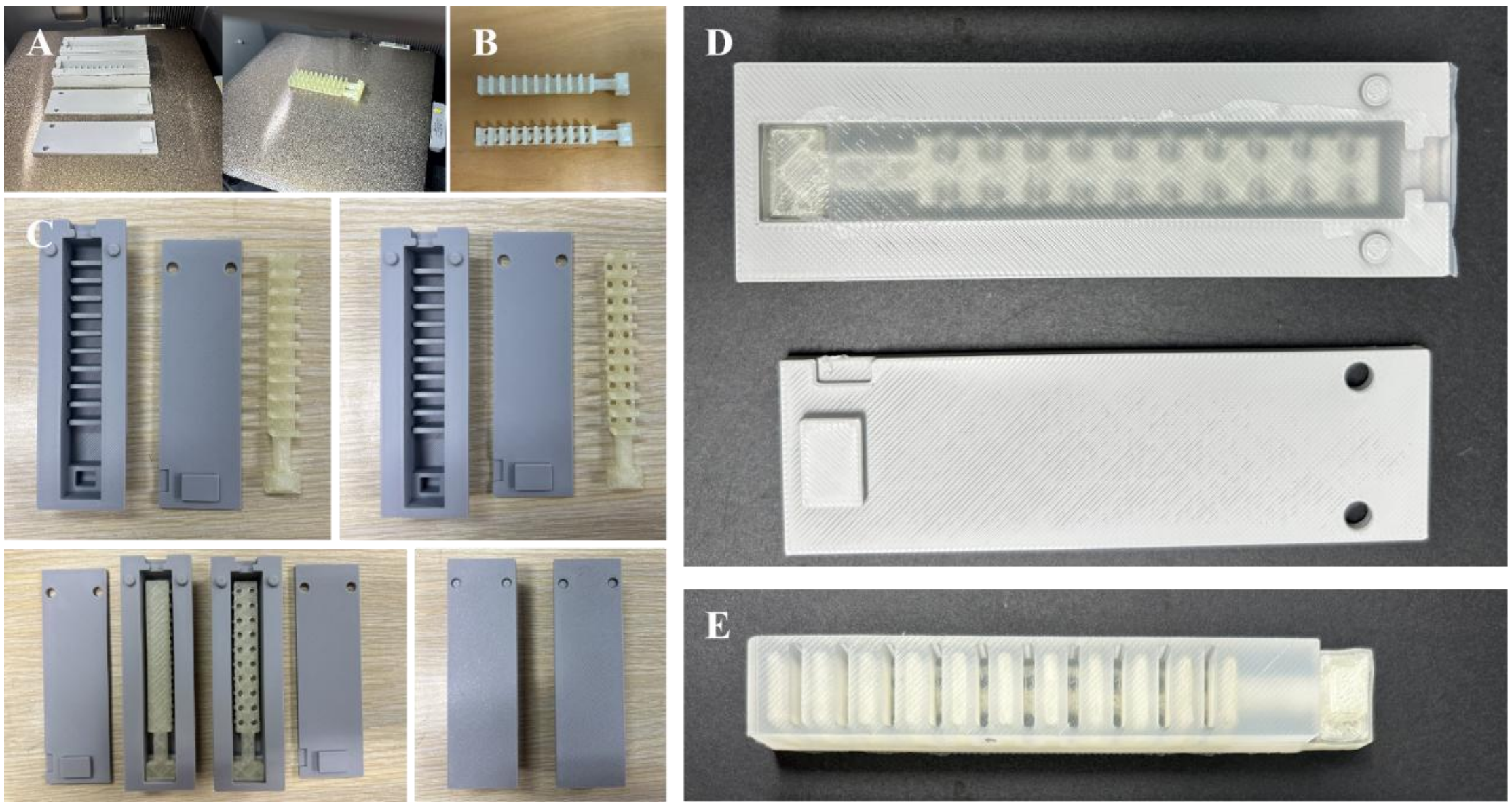

Fig. S4. Sacrificial-core casting process before PVA dissolution. (A) PLA-printed molds and water-soluble PVA sacrificial core used for MARS fabrication. (B) Comparison of PVA sacrificial cores for U-SPA and MARS, showing the column-free core for U-SPA and the column-forming core for MARS. (C) Mold components and assembled casting configurations. (D) Silicone body cured around the embedded PVA sacrificial core within the opened mold. (E) Demolded as-cast MARS actuator containing the PVA sacrificial core before sacrificial-core dissolution.

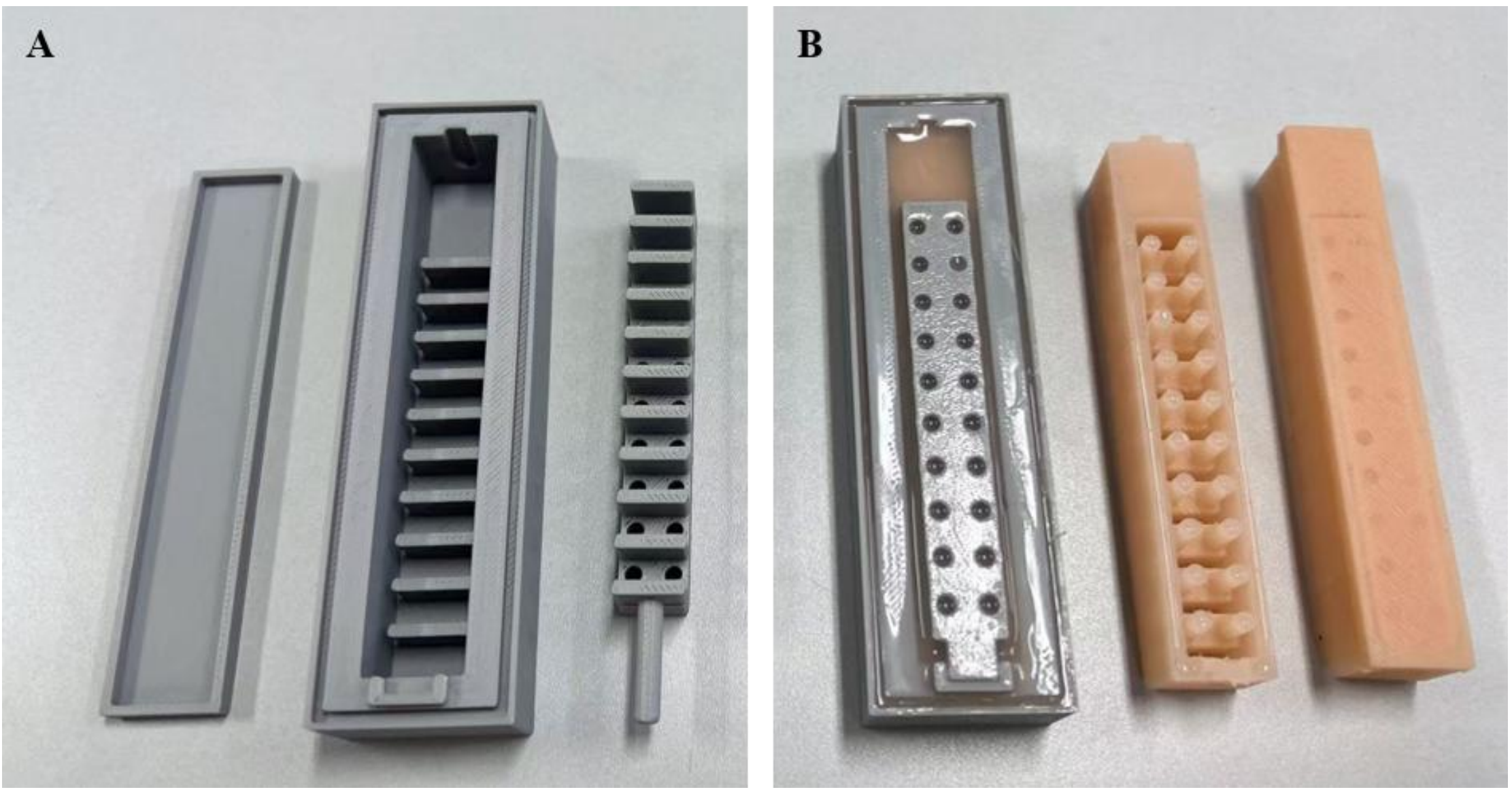

Fig. S5. Conventional multistep fabrication of MARS. (A) Mold components used for conventional fabrication. (B) Fabrication states after the first curing step in the mold, after demolding before secondary curing, and after secondary curing to obtain the complete MARS actuator.

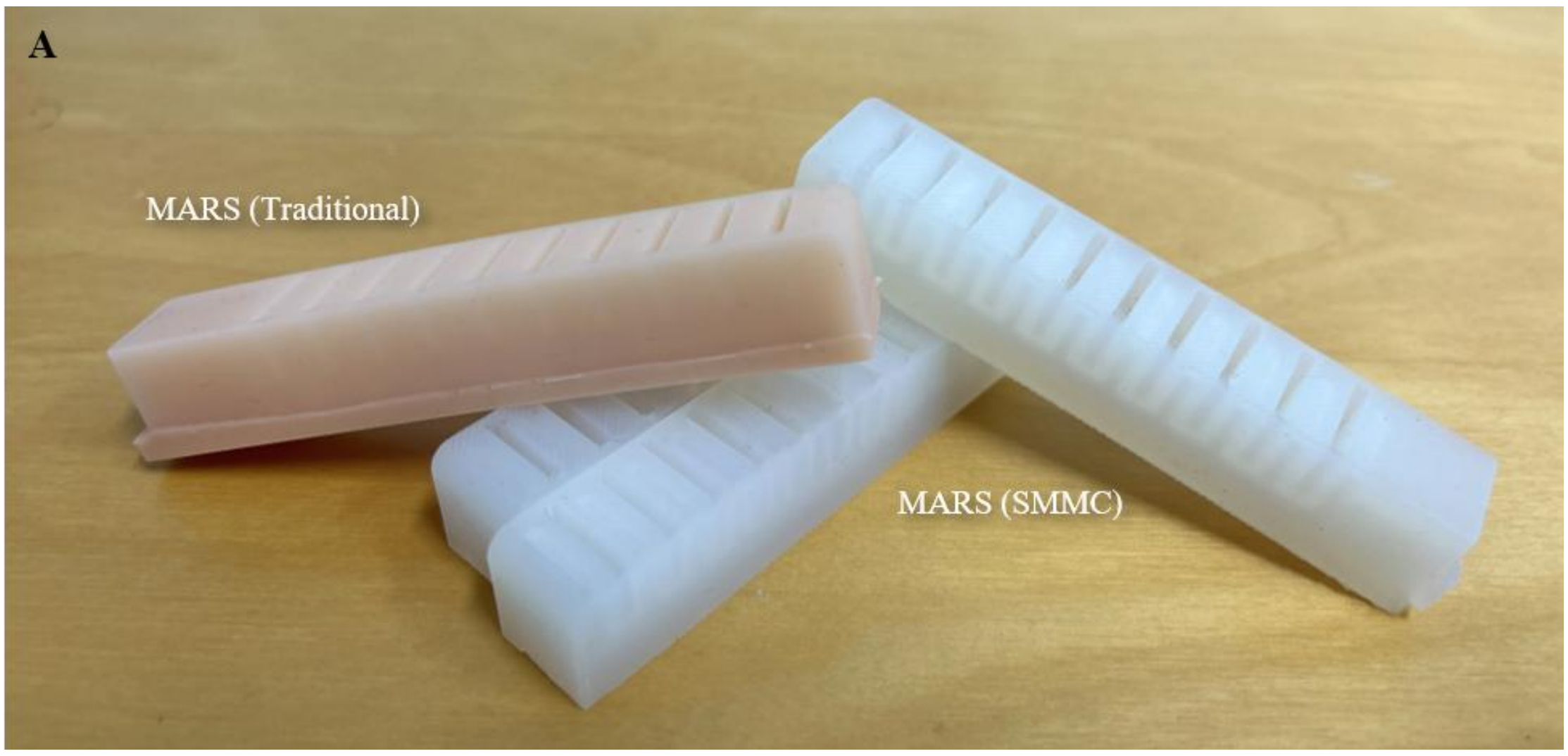

Fig. S6. Prototype exhibition of MARS actuators. Representative MARS prototypes fabricated by conventional molding and SMMC are shown to illustrate the appearance of the fabricated actuators.

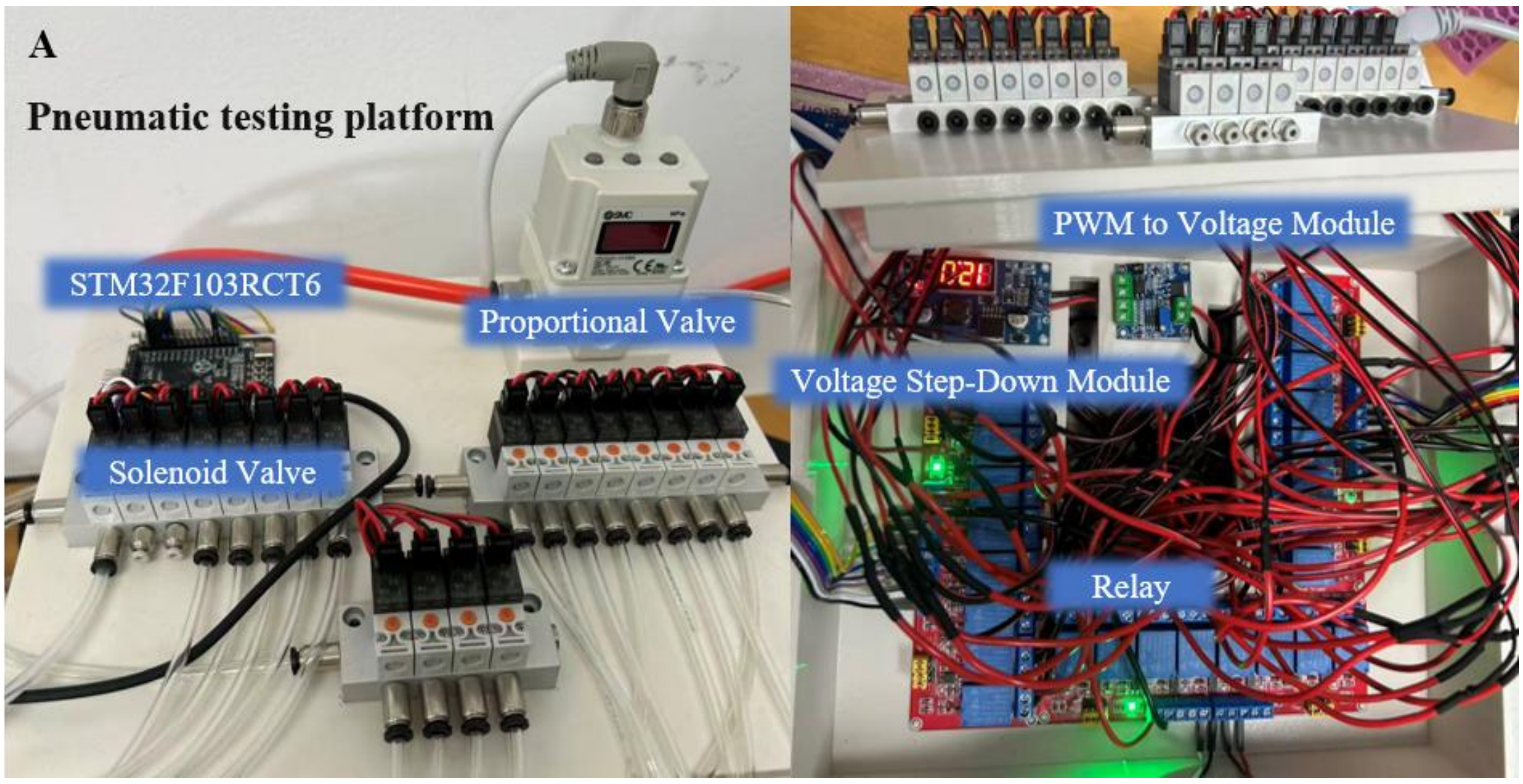

Fig. S7. Twenty-channel pneumatic testing platform. The twenty-channel pneumatic testing platform was controlled using an STM32F103RCT6 microcontroller. The microcontroller operated 20-channel relay modules connected to 20-channel solenoid valve manifolds (D271-1, Zhejiang Keyihua Pneumatic Technology Co., Ltd., China). Regulated positive pressure was supplied by an air compressor (15L-1760, Taizhou Ortus Industrial and Trade Co., Ltd., China) connected to proportional valves (ITV1031-312, SMC Corp., Japan). Silicone tubing (Hangzhou Kaiji Trading Co., Ltd., China) was used to connect the pneumatic system to the actuators.

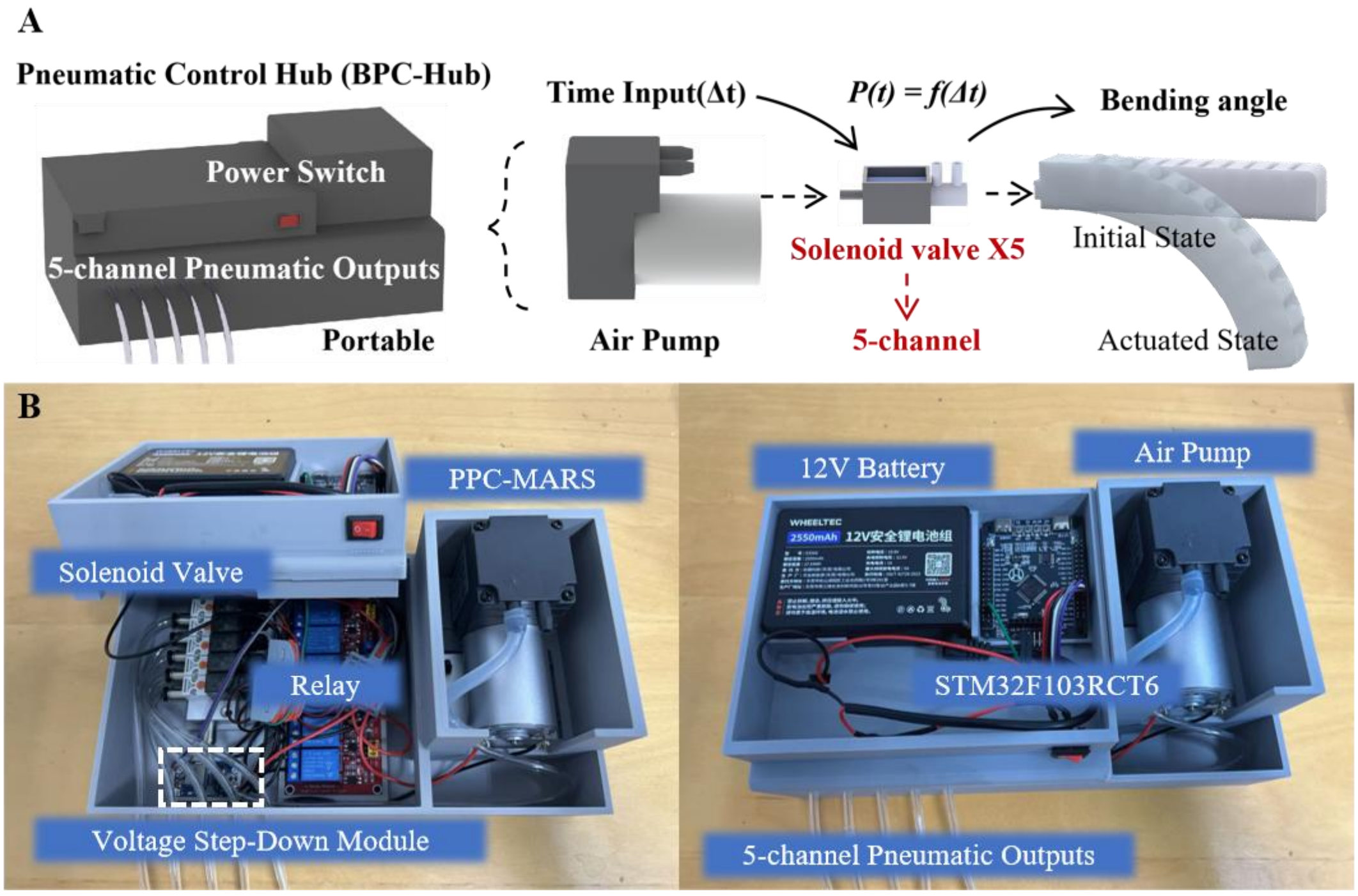

Fig. S8. Portable pneumatic controller (PPC) for MARS actuation. (A) A compact controller with multi-channel pneumatic outputs enables pressure-regulated operation of the MARS actuator. (B) PPC integrated a 12 V battery, STM32F103RCT6 microcontroller, relay modules, solenoid valves, voltage step-down module, air pump, and five pneumatic output channels. The microcontroller controlled the relay-driven solenoid valves to route air pressure from the pump to selected MARS actuators. The actuator pressure was regulated by the valve-opening duration, and application-specific control codes were used to generate pneumatic sequences for different robotic tasks.

A

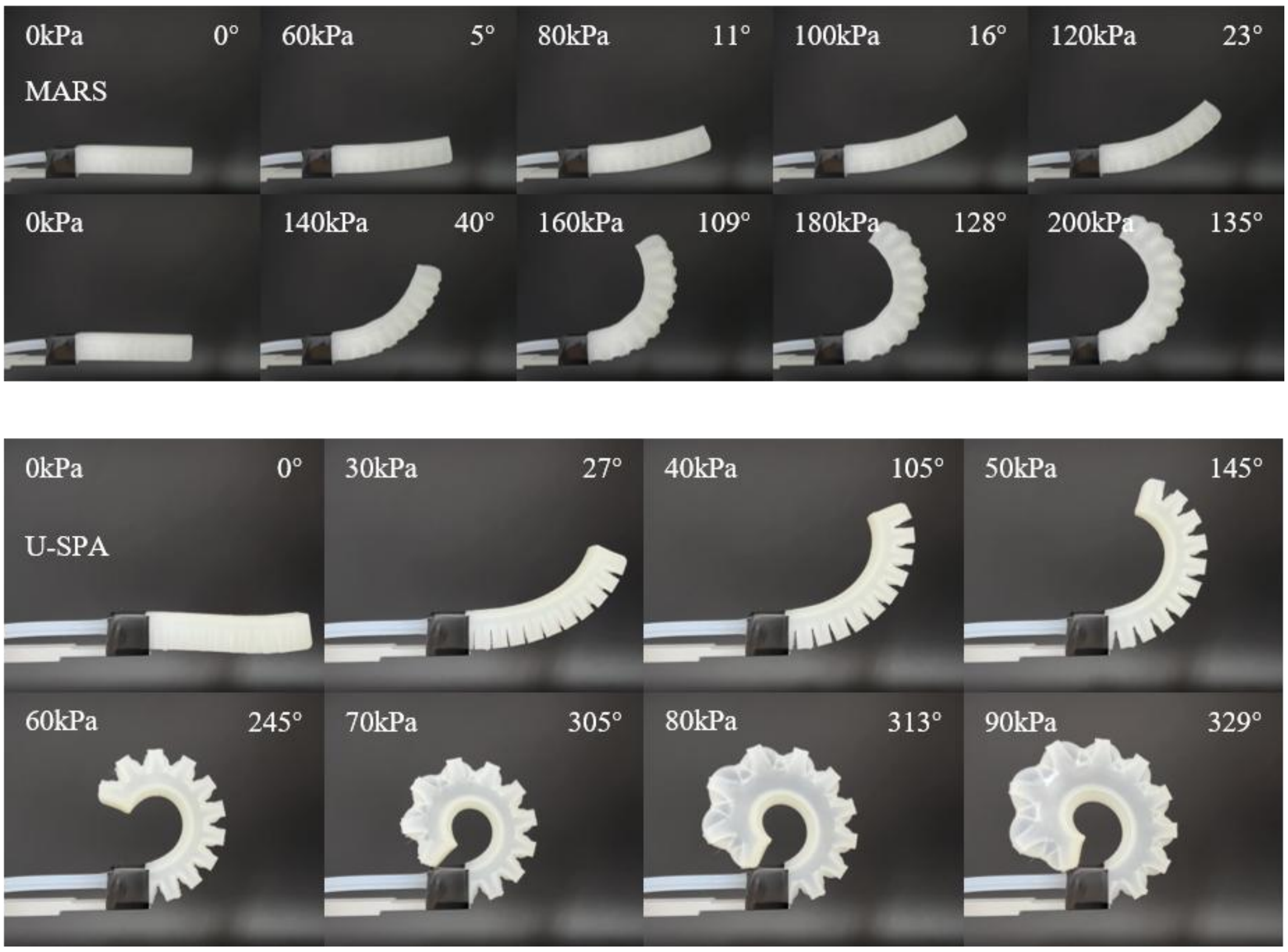

Fig. S9. Pressure-dependent deformation of MARS and U-SPA actuators. (A) Deformational behavior of the MARS actuator, which demonstrates high structural stability and graduates to a maximum bending angle of 135° at an operational pressure of 200 kPa. (B) Bending performance of the U-SPA actuator, which exhibits a highly sensitive response at lower pressures, reaching a large-angle curling of 329° at 90 kPa along with noticeable local material expansion at higher pressure ranges.

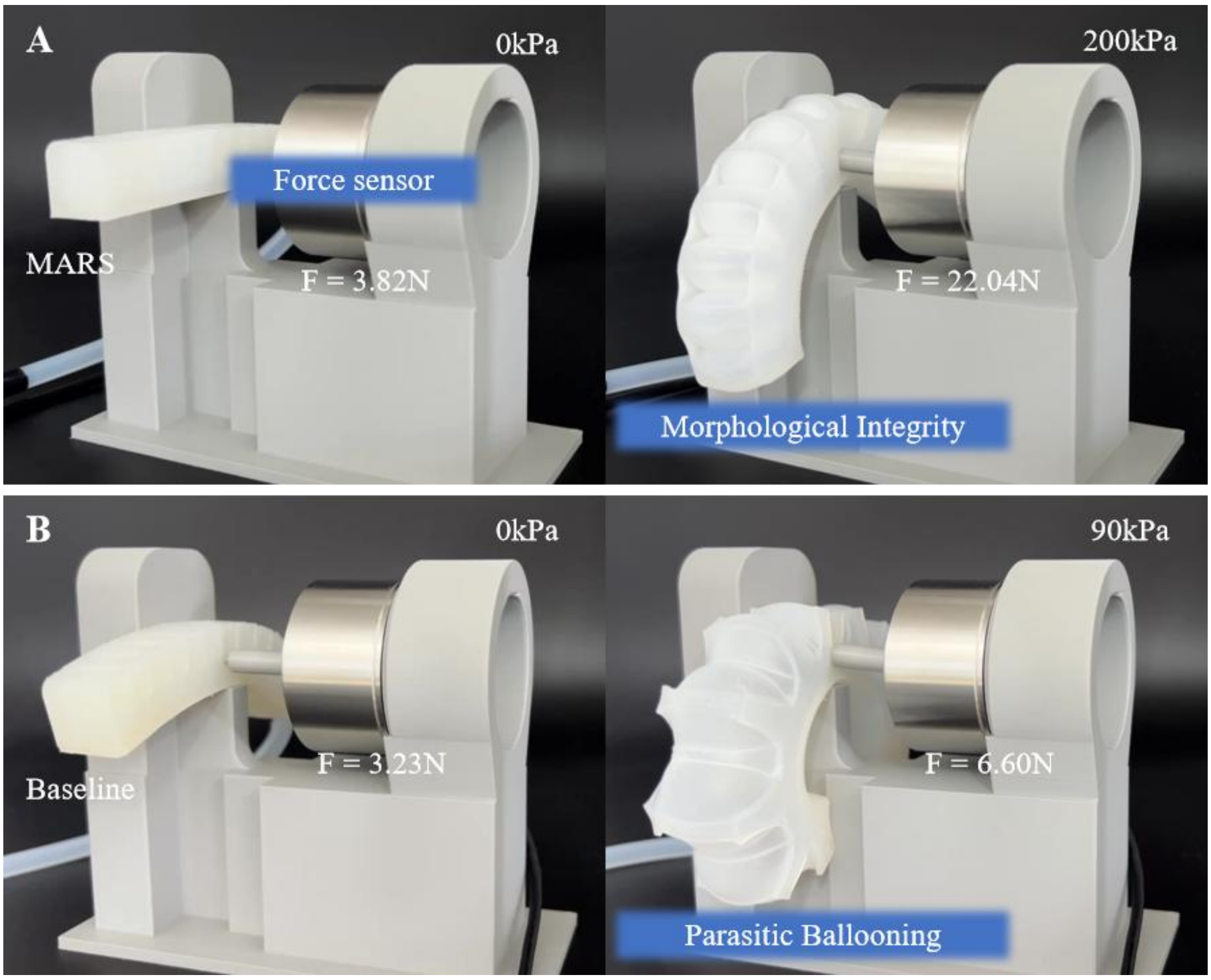

Fig. S10. Local lateral indentation stiffness characterization under pressurization. (A and B) Experimental setup and representative deformation states of MARS and Baseline. A force-sensing probe was applied to the mid-body region of the actuator with a fixed lateral indentation displacement of Δx=2 mm. for all measurements. The lateral indentation stiffness was calculated as $K_{lat}=F/\Delta x$. where F is the measured reaction force. The mid-body region was selected to provide stable specimen positioning and consistent probe contact during large-deformation actuation. (A) MARS exhibited an increase in lateral indentation stiffness from 1.91 N/mm at 0 kPa to 11.02 N/mm at 200 kPa while maintaining its overall morphology. (B) Baseline showed a smaller stiffness increase, reaching 3.30 N/mm at 90 kPa, accompanied by radial ballooning.

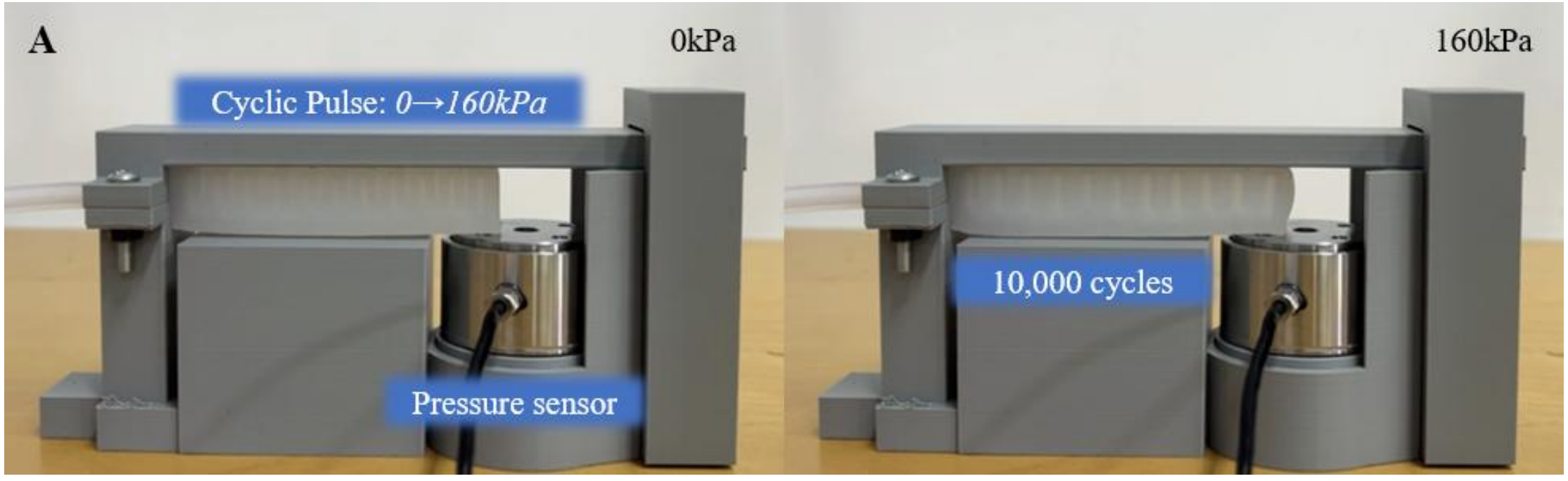

Fig. S11. Configuration of the constrained fingertip-force and cyclic durability tests. (A) Snapshots of the MARS actuator in the resting state at 0 kPa and under pressurization at 160 kPa. The actuator was cyclically driven between 0 and 160 kPa for 10,000 cycles, with each cycle consisting of 1200 ms inflation and 800 ms deflation. The same fixture was used for constrained fingertip-force measurements and cyclic durability testing.

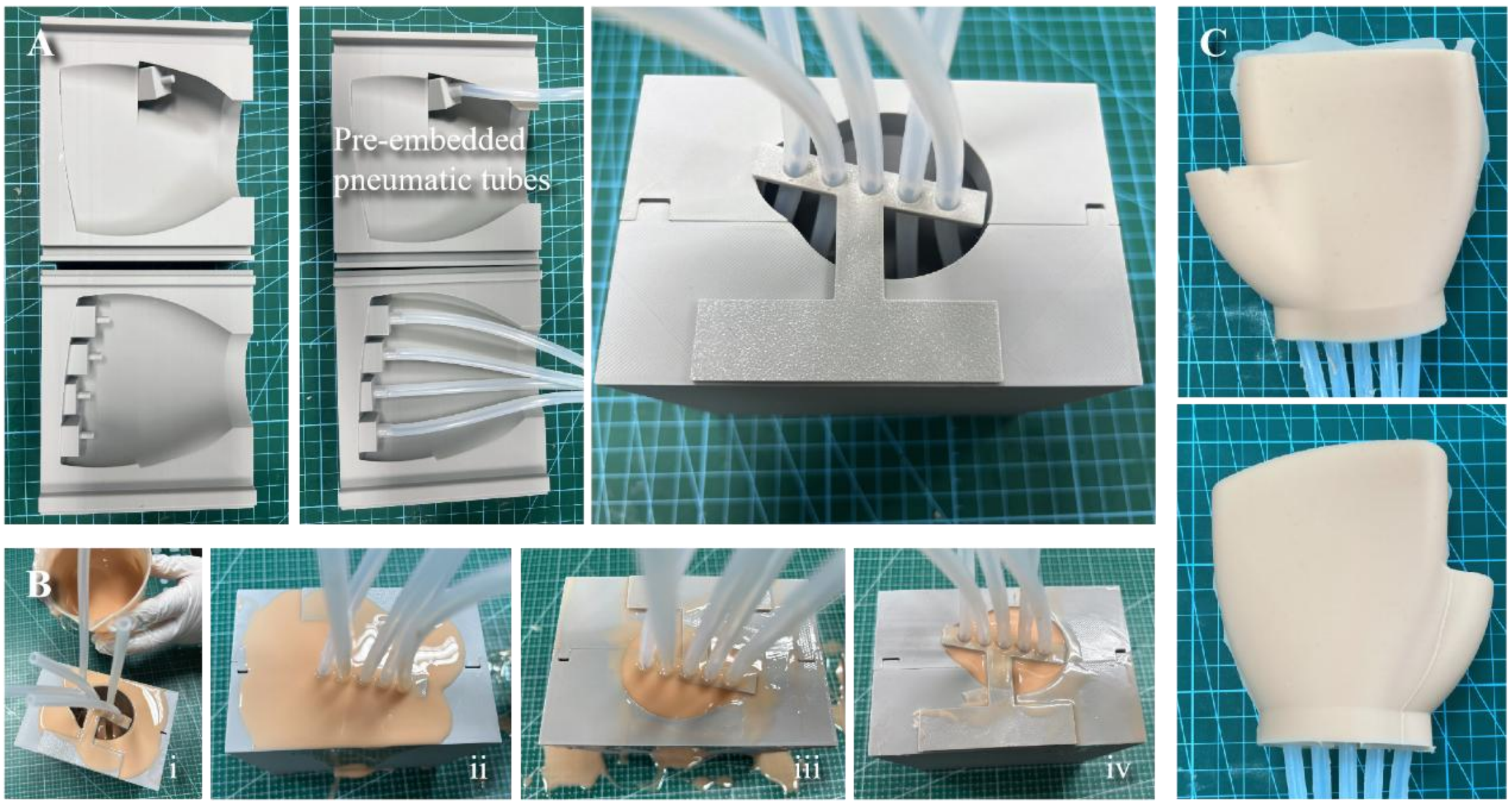

Fig. S12. Mold casting of the silicone palm with pre-embedded pneumatic tubes. (A) Mold design and assembly for the MARS-DexHand palm, including the PLA-printed palm mold, pre-embedded pneumatic tubes, and the closed mold configuration. (B) Stepwise casting process: (i) skin-toned silicone was poured into the assembled mold, (ii) the mold was filled until silicone overflowed, (iii) excess silicone was scraped from the mold surface, and (iv) the silicone was cured in the mold. (C) Demolded silicone palm containing the integrated pneumatic tubes, shown from the front and back sides.

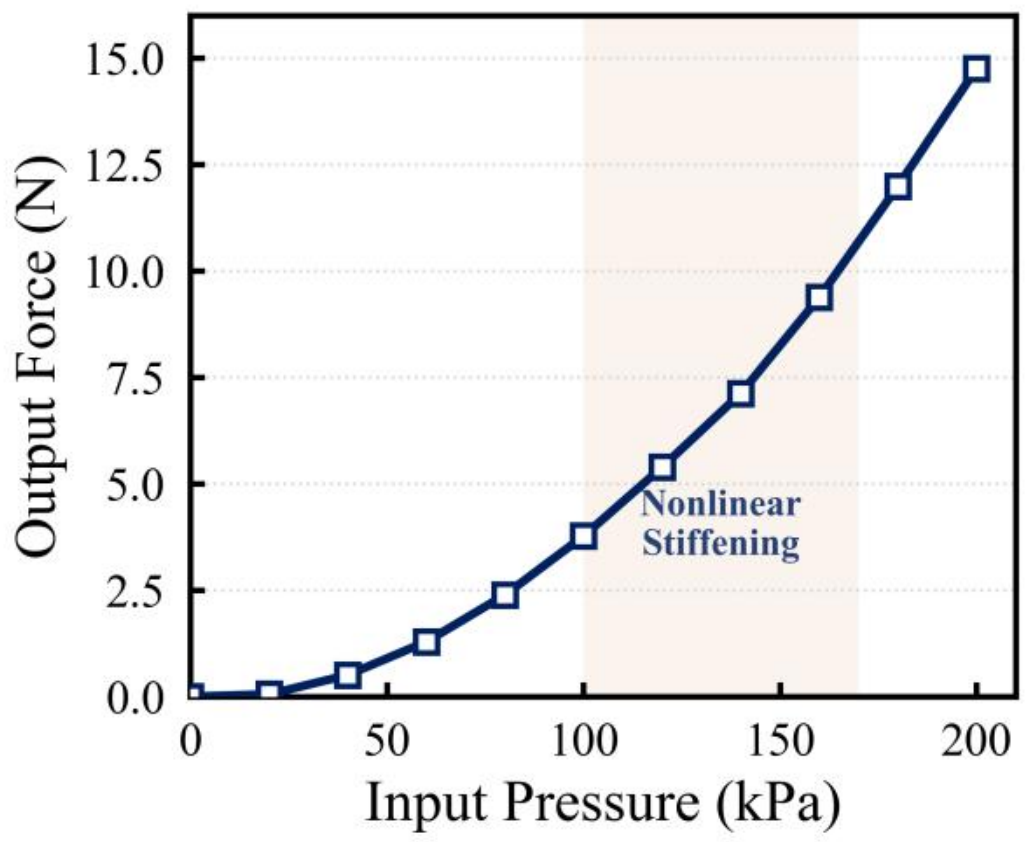

Fig. S13. Pressure-dependent fingertip force generation of the MARS-enhanced actuator. The output force increases continuously with increasing pneumatic pressure and reaches a maximum fingertip force of approximately 14.8 N at 200 kPa, demonstrating the enhanced force generation capability of the MARS actuator.

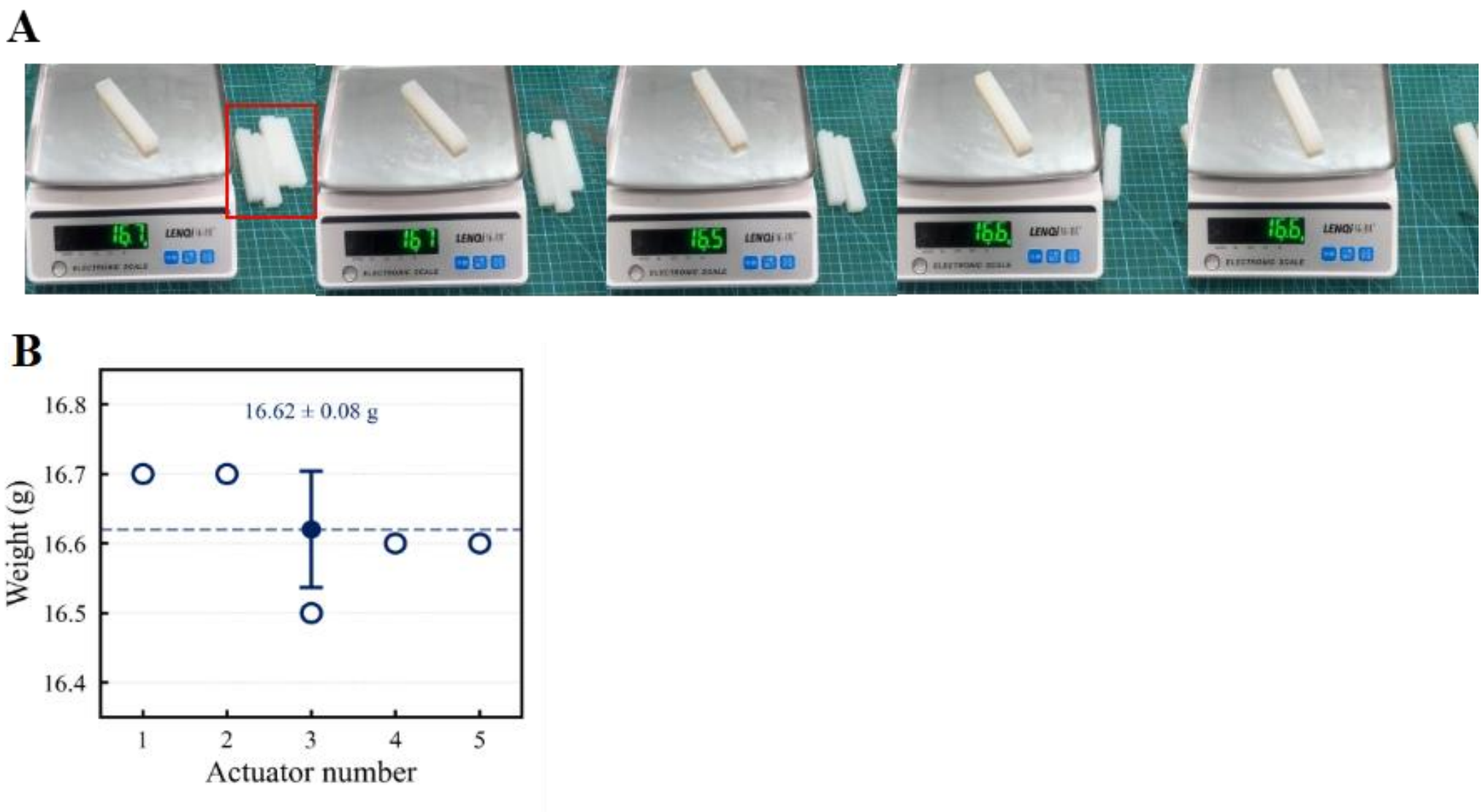

Fig. S14. Fabrication consistency of independently produced MARS-enhanced actuators. (A) Photographs of five independently fabricated MARS-enhanced actuators and their measured weights. (B) Statistical analysis of actuator weight variation, showing an average weight of 16.62 ± 0.08 g across five samples, indicating high fabrication consistency.

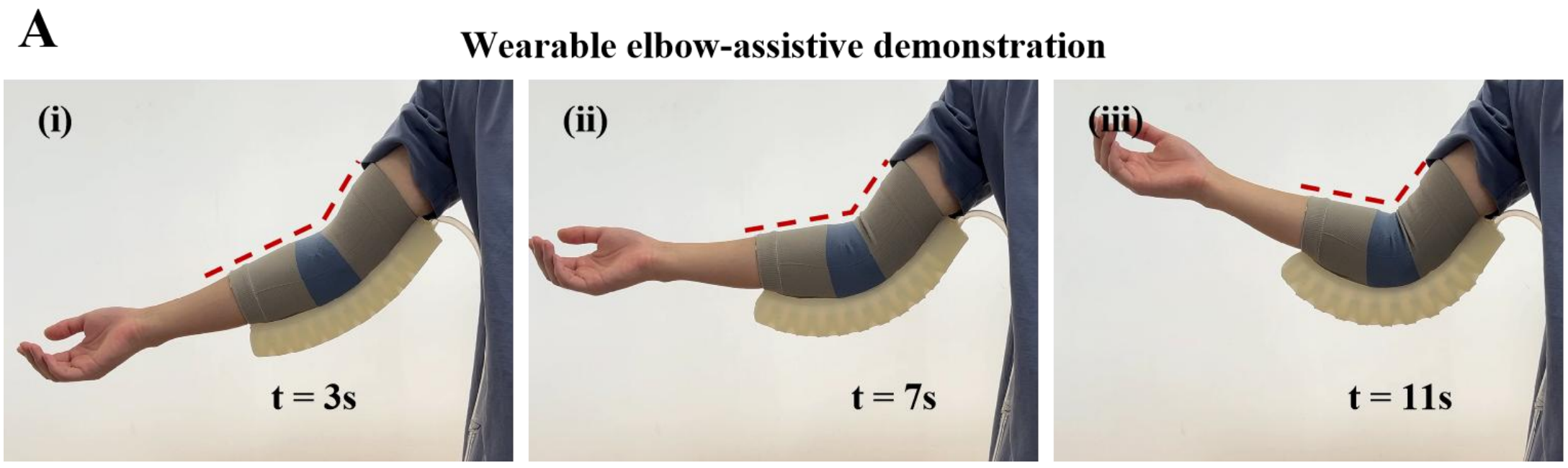

Fig. S15. Scalability demonstration of the MARS-enhanced actuator for human-scale applications. A 2.5× enlarged MARS actuator was fabricated while maintaining the original architectural design. The enlarged actuator was integrated into a wearable sleeve and demonstrated continuous elbow flexion assistance, highlighting the scalability of the MARS architecture toward larger human–machine interaction scenarios.

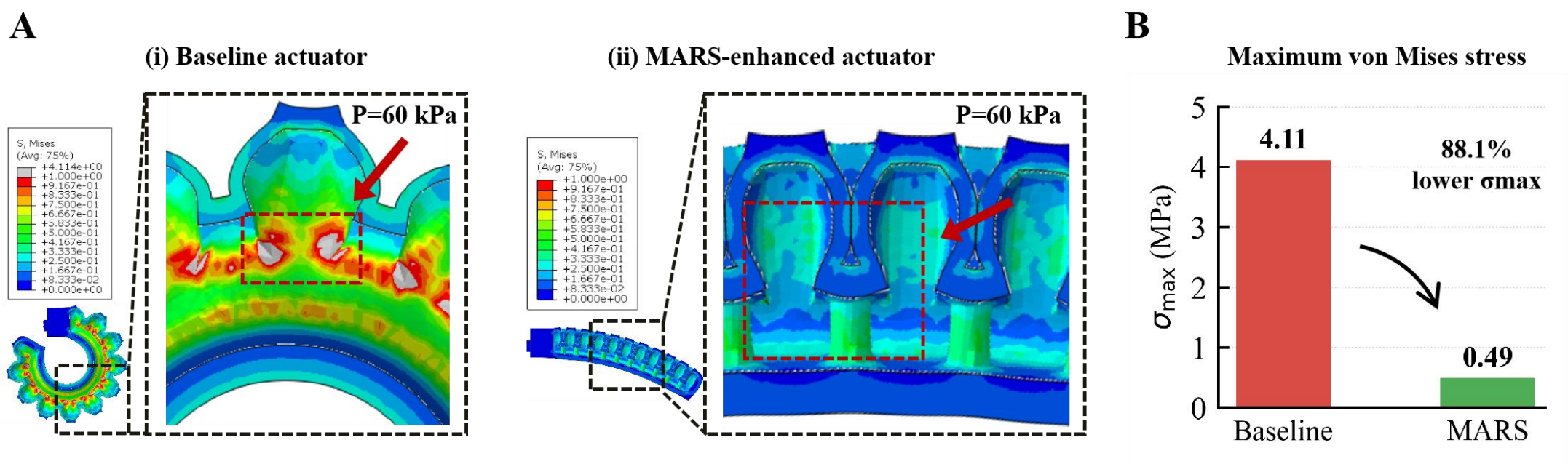

Fig. S16. Finite element analysis of stress distribution for baseline and MARS-enhanced actuators. (A) Von Mises stress distributions of (i) the baseline actuator without internal reinforcement and (ii) the MARS-enhanced actuator with internal beam–column load paths under 60 kPa pneumatic pressure. The baseline actuator exhibits localized stress concentration, whereas the MARS-enhanced actuator enables stress redistribution through the internal architecture. (B) Comparison of the maximum von Mises stress between the two actuators, showing a reduction from 4.11 MPa to 0.49 MPa after introducing the MARS architecture.

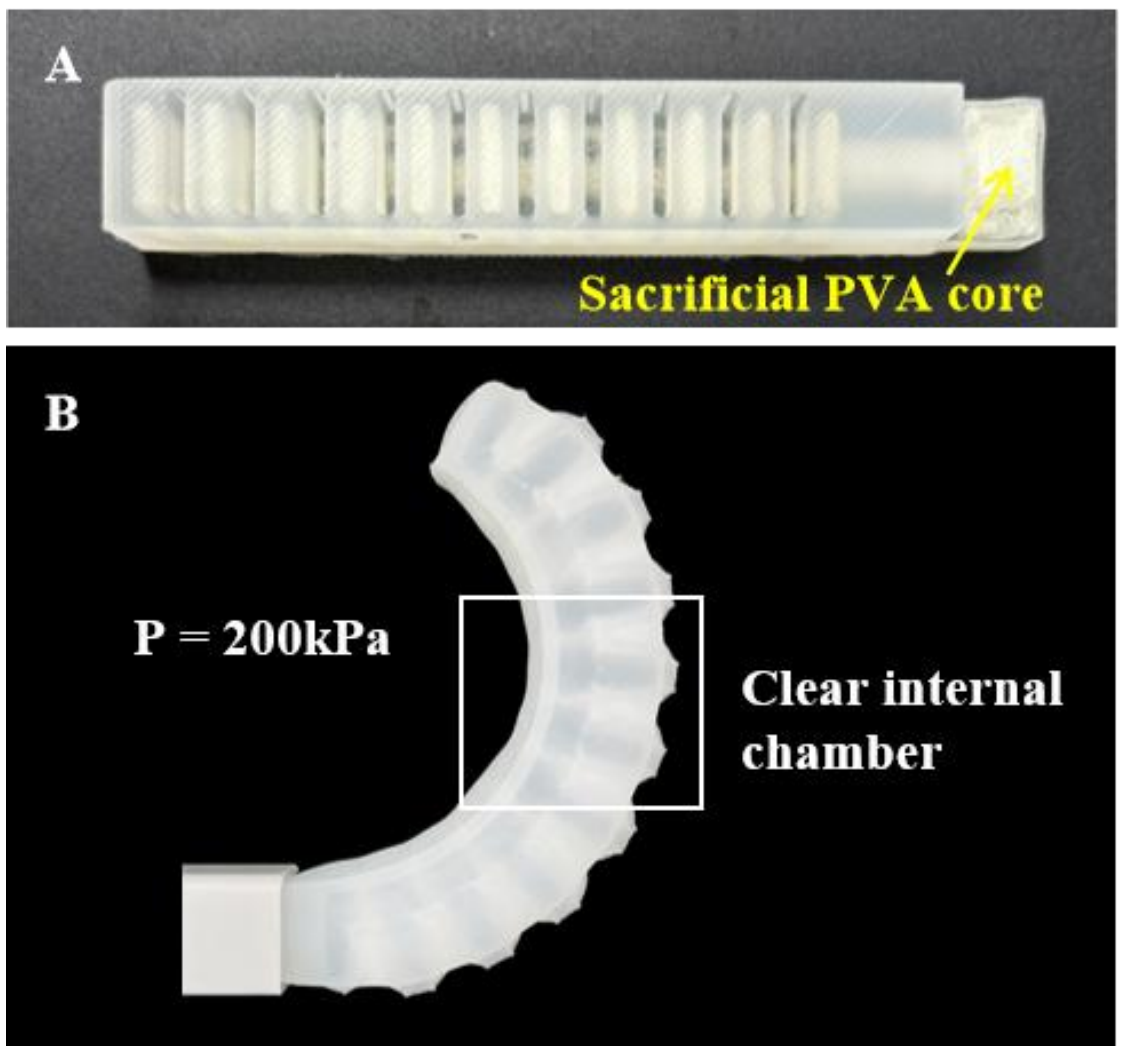

Fig. S17. Removal of the PVA sacrificial core during fabrication of the MARS actuator. (A) Actuator containing the PVA sacrificial core before dissolution. (B) Actuator after PVA dissolution and pneumatic inflation at 200 kPa, showing the formed internal chamber.

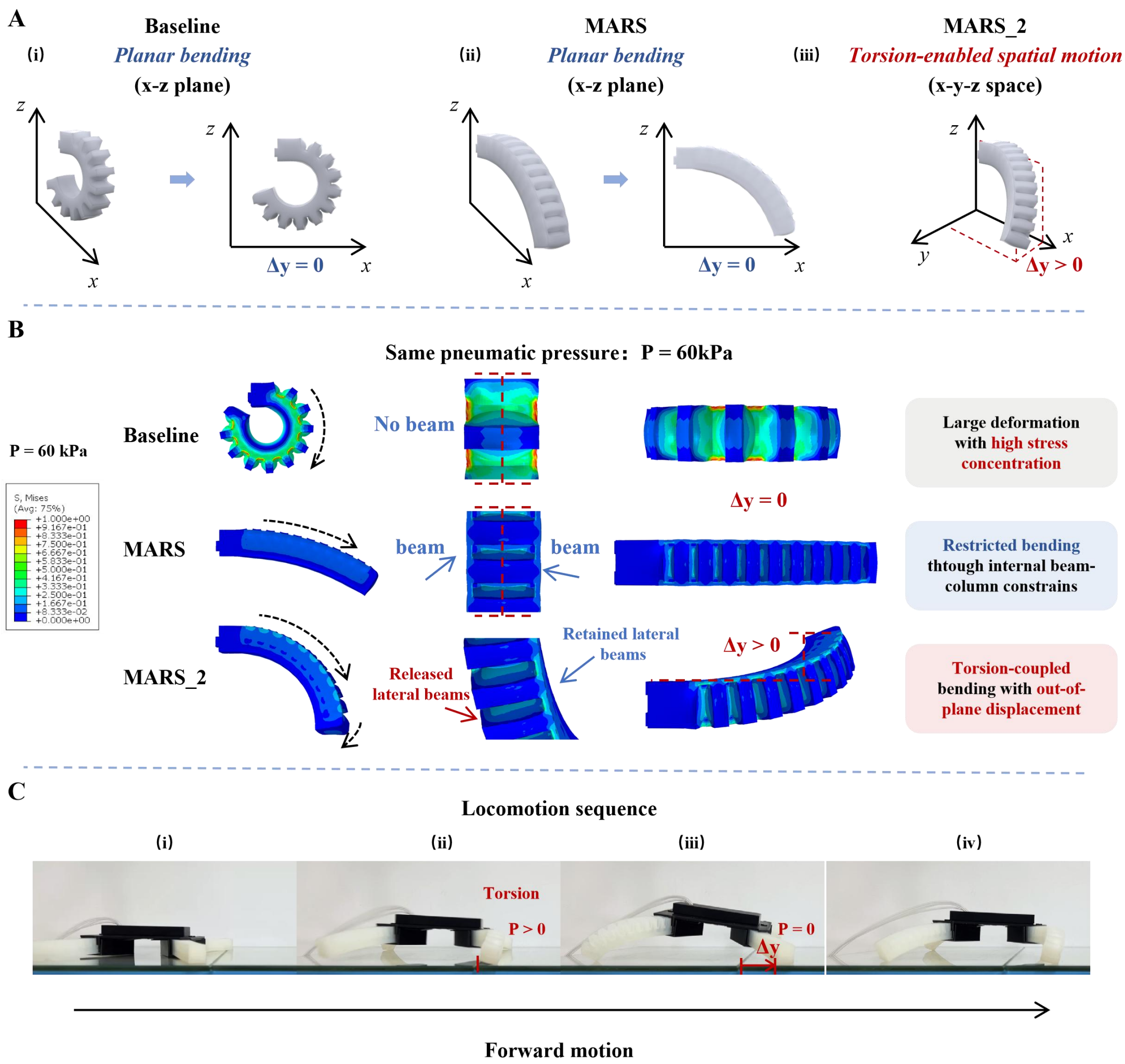

Fig. S18. Mechanistic comparison of baseline, MARS, and MARS_2 actuators for locomotion. (A) Comparison of deformation modes. The baseline and intact MARS actuators primarily exhibit planar bending in the x-z plane, whereas MARS_2 generates torsion-enabled spatial motion with an out-of-plane displacement ($\Delta y > 0$). (B) Finite-element comparison under the same pneumatic pressure ($P = 60$ kPa). The baseline actuator exhibits large deformation and localized stress concentration, while the internal beam–column constraints in MARS regulate bending. Asymmetric release of the lateral beams in MARS_2 breaks the symmetric constraint and generates torsion-coupled bending with an out-of-plane component. (C) Representative locomotion sequence showing the conversion of torsional limb actuation and recovery into forward motion.

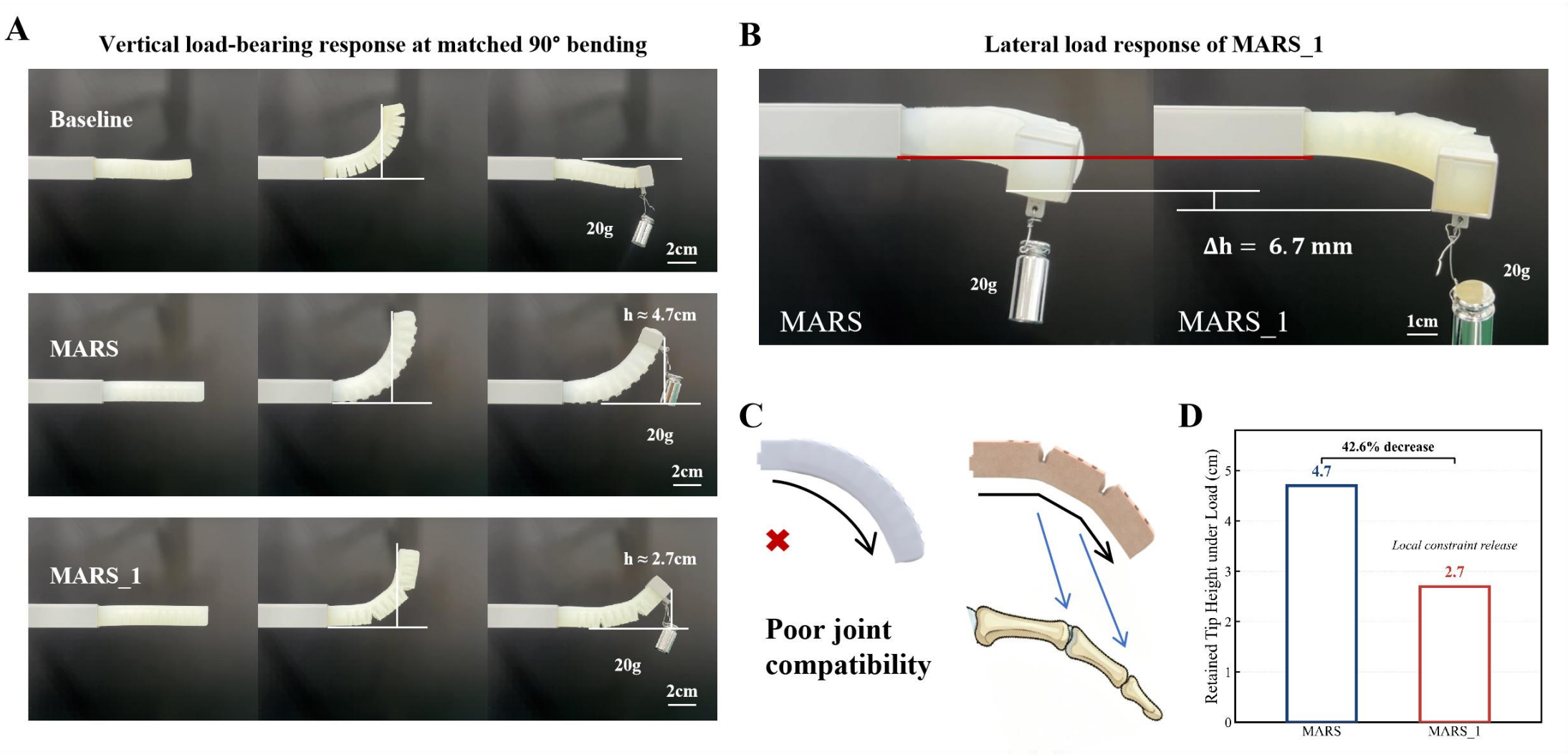

Fig. S19. Mechanical trade-off associated with localized beam removal in MARS. (A) Load-bearing deformation of the baseline, MARS, and MARS_1 actuators. The actuators were first adjusted to an approximately 90° bending configuration and subsequently subjected to the same 20 g vertical load. MARS retained stronger structural support, whereas MARS_1 exhibited increased deformation after local beam removal. (B) Lateral-load response of MARS_1 under a 20 g transverse load, showing a lateral displacement of approximately 6.7 mm. (C) Schematic comparison of continuous bending in intact MARS and localized hinge-like bending in MARS_1 illustrating how local constraint release improves compatibility with finger-joint kinematics. (D) Quantification of the retained tip height under the 20 g vertical load. The retained height decreased from approximately 4.7 cm for MARS to 2.7 cm for MARS_1, corresponding to a 42.6% reduction.

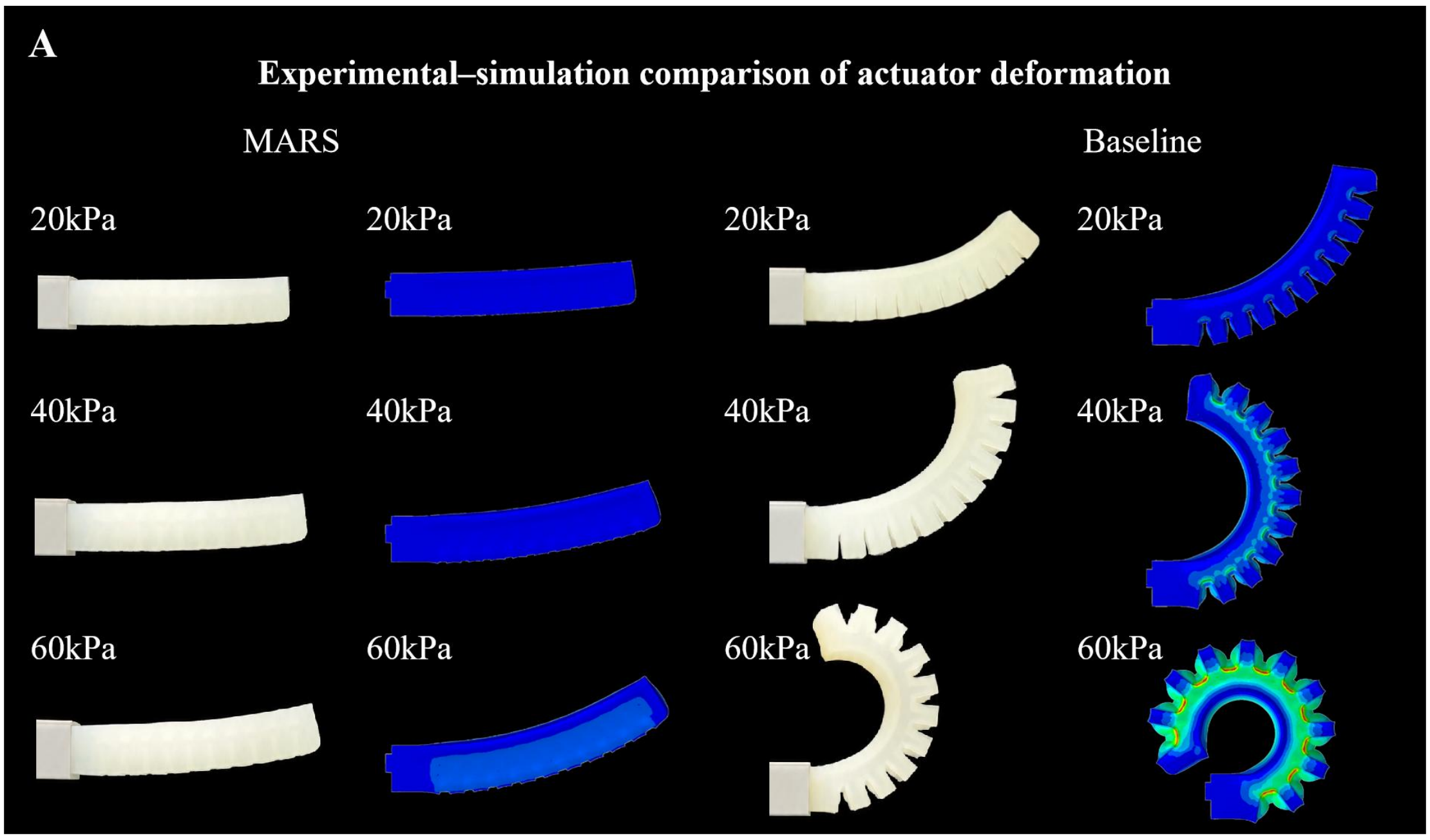

Fig. S20. Experimental–simulation comparison of pressure-dependent actuator deformation. Experimental and simulated deformation of MARS and baseline actuators under 20, 40, and 60 kPa pneumatic pressures. The simulations capture the deformation evolution and structural constraints introduced by the embedded beam–column architecture, consistency with the experimentally observed bending trends.

Table S1. Comparison of MARS with representative stiffness-enhancement strategies in soft robotic systems

Reference	Body-integrated materials (n)	Compliance preservation	Auxiliary stiffening components	Reported stiffness enhancement	Motion programmability
**This work**	**1**	**Yes**	**No**	**5.8-fold lateral stiffness increase**	**Post-defined**
(8)	4	No	Yes	53-fold apparent bending modulus	Predesigned
(46)	4	No	Yes	>20-fold tensile stiffness	Reprogrammable
(47)	≥ 5	No	Yes	~5-fold reversible stiffening	Limited
(48)	≥ 5	No	Yes	21.3 jamming ratio	Limited
(49)	≥ 5	No	Yes	~8-fold overall stiffness	Mode-dependent operation
(50)	≥ 5	No	Yes	~7-fold vacuum-state stiffness	Limited

*Note: Material systems were counted only for body-integrated materials. Materials used only for tubing, controllers, connectors, or external supply units were excluded. Adhesives were counted only when they formed part of the robot or actuator body.

Table S2A. Environmental breadth scores for Tardigrade-Bot and representative soft robotic systems

Ref.	Robot/system	E1	E2	E3	E4	E5	E6	E7	Env
**This work**	**Tardigrade-Bot**	**1**	**1**	**1**	**1**	**1**	**1**	**1**	**7/7**
(34)	Magnetic quadruped soft microrobot	1	1	0	0	0	1	0	3/7
(35)	Electrohydraulic amphibious robot	1	0	0	1	1	0	0	3/7
(36)	Wireless amphibious origami millirobot	1	1	0	1	0	1	0	4/7
(37)	Ultra-thin TS-Robot	1	1	0	1	1	1	0	5/7
(38)	Amphibious Robotic Turtle	1	1	1	1	1	0	0	5/7
(39)	Growth-based soft robot	1	1	0	0	0	1	0	3/7
(40)	Electronics-free soft-legged robot	1	0	0	0	0	1	0	2/7

Binary scores: 1 = criterion satisfied; 0 = criterion not satisfied. Env is the sum of E1–E7. Scores use only same-platform experimental evidence reported in this work and refs. *34–40*.

Table S2B. Functional integration scores for Tardigrade-Bot and representative soft robotic systems

Ref.	Robot/system	F1	F2	F3	F4	F5	F6	F7	Func	Total
**This work**	**Tardigrade-Bot**	**1**	**1**	**1**	**1**	**1**	**1**	**1**	**7/7**	**14/14**
(34)	Magnetic quadruped soft microrobot	1	1	1	1	1	0	0	5/7	8/14
(35)	Electrohydraulic amphibious robot	0	1	1	0	0	0	1	3/7	6/14
(36)	Wireless amphibious origami millirobot	0	1	1	1	1	1	0	5/7	9/14
(37)	Ultra-thin TS-Robot	0	1	1	1	0	0	0	3/7	8/14
(38)	Amphibious Robotic Turtle	1	1	1	0	0	0	0	3/7	8/14
(39)	Growth-based soft robot	0	0	1	0	1	0	0	2/7	5/14
(40)	Electronics-free soft-legged robot	1	0	1	0	1	0	0	3/7	5/14

Binary scores: 1 = criterion satisfied; 0 = criterion not satisfied. Func is the sum of F1–F7; Total = Env + Func, using the corresponding Env score in Table S2A.

Detailed scoring rules for Table S2

General evidence rule. Each criterion was scored conservatively as 0 or 1. A score of 1 was assigned only when the capability was experimentally demonstrated by the same robot platform. Review statements, proposed future functions, single-actuator tests, simulations without robot-level validation, and capabilities assembled from different robots in the same article were not counted.

Zero-score rule. A score of 0 indicates that qualifying same-platform experimental evidence was not identified in the evaluated article; it does not prove that the robot is fundamentally incapable of that function.

Environmental breadth (E1–E7)

Code	Criterion	A score of 1 requires
**E1**	Flat terrestrial locomotion	Continuous locomotion on a flat solid surface.
**E2**	Non-flat terrain locomotion	Locomotion on a slope, curved surface, pipe, step, vertical surface, or another clearly non-flat terrain.
**E3**	Deformable/slippery natural substrates	Locomotion on at least three natural, unstructured, deformable, wet, slippery, granular, or vegetated substrates.
**E4**	Aquatic locomotion	Experimentally demonstrated locomotion underwater, on the bottom, or at the water surface.
**E5**	Cross-medium transition	A continuous experimental transition across land-water, water-air, or another genuine medium interface. Aquatic locomotion alone does not qualify.
**E6**	Confined/obstructed environment	Locomotion in a narrow, obstructed, high-contact, or otherwise constrained environment.
**E7**	Out-of-lab/field deployment	Operation in an outdoor or realistic unstructured setting using portable, onboard, wireless, or otherwise field-deployable actuation/control.

Functional integration (F1–F7)

Code	Criterion	A score of 1 requires
**F1**	Body-elevated soft locomotion	The robot body is elevated and supported by soft limbs or soft structures rather than being fully dragged along the substrate.
**F2**	Multimodal locomotion	The same robot platform experimentally performs at least two distinct locomotion modes.
**F3**	Same-platform operation	The scored capabilities are demonstrated by the same robot platform without replacing the robot body or primary actuators.
**F4**	Payload-bearing locomotion	The robot continues to move while carrying an added payload. Static load support alone does not qualify.
**F5**	Portable/onboard actuation or control	Actuation or control is portable tethered, onboard, wireless, or otherwise deployable outside a fixed laboratory installation.
**F6**	Recovery/escape-like capability	The robot continues, recovers, or escapes after obstruction, slipping, entrapment, entanglement, or confinement. Autonomous self-rescue is not implied unless sensing and decision-making are demonstrated.
**F7**	Environmental robustness test	Robot-level locomotion is experimentally retained under temperature, wet/slippery, endurance, cyclic, or another relevant environmental or operational stress.

Interpretive safeguards. For F4, static load support was not counted as payload-bearing locomotion. For E5, aquatic locomotion alone was not counted as a cross-medium transition. Wireless or untethered actuation alone did not automatically qualify for E7. F6 is described as recovery/escape-like capability unless autonomous sensing and decision-making were explicitly demonstrated.
