# Supplementary material for: Architected Load Paths Enable Stiffening and Motion Programming in Soft Pneumatic Actuators
Source: Adv Sci (Weinh). 2026 Sep 27:e77868. Online ahead of print. doi: 10.1002/advs.77868 (PMC13616343; doi:10.1002/advs.77868)
Supplement: Supplementary file 2 [file ADVS-9999-e77868-s005.docx]

- This work introduces a monolithic architected reinforcement strategy that embeds load-bearing paths within soft pneumatic actuators.

- Architected load paths redirect pneumatic loading to suppress ballooning while enhancing stiffness and force output.

- MARS-enhanced actuators achieve a 5.8-fold increase in lateral stiffness and generate up to 8.4-fold greater tip force.

- The same actuator architecture can be post-programmed to produce continuous bending, localized joint-like bending, and spatial torsion.
